# APOBEC-1 deletion enhances cisplatin-induced acute kidney injury

**DOI:** 10.1038/s41598-023-49575-3

**Published:** 2023-12-14

**Authors:** Xiaojia Guo, Valerie Blanc, Nicholas O. Davidson, Heino Velazquez, Tian-min Chen, Dennis G. Moledina, Gilbert W. Moeckel, Robert L. Safirstein, Gary V. Desir

**Affiliations:** 1 Section of Nephrology, Department of Internal Medicine, Yale School of Medicine, New Haven, CT USA; 2 Division of Gastroenterology, Department of Medicine, Washington University School of Medicine, St. Louis, MO 63105 USA; 3 Veteran’s Affair Medical Center, West Haven, CT USA; 4 Clinical and Translational Research Accelerator, Department of Internal Medicine, Yale School of Medicine, New Haven, CT USA; 5 Pathology, Yale School of Medicine, New Haven, CT USA

**Keywords:** Cell biology, Immunology, Molecular biology, Physiology, Diseases, Medical research, Nephrology, Pathogenesis

## Abstract

Cisplatin (CP) induces acute kidney injury (AKI) whereby proximal tubules undergo regulated necrosis. Repair is almost complete after a single dose. We now demonstrate a role for Apolipoprotein B mRNA editing enzyme, catalytic polypeptide 1 (Apobec-1) that is prominently expressed at the interface between acute and chronic kidney injury (CKD), in the recovery from AKI. Apobec-1 knockout (KO) mice exhibited greater mortality than in wild type (WT) and more severe AKI in both CP- and unilateral ischemia reperfusion (IR) with nephrectomy. Specifically, plasma creatinine (pCr) 2.6 ± 0.70 mg/dL for KO, n = 10 and 0.16 ± 0.02 for WT, n = 6, p < 0.0001 in CP model and 1.34 ± 0.22 mg/dL vs 0.75 ± 0.06, n = 5, p < 0.05 in IR model. The kidneys of Apobec-1 KO mice showed increased necrosis, increased expression of KIM-1, NGAL, RIPK1, ASCL4 and increased lipid accumulation compared to WT kidneys (p < 0.01). Neutrophils and activated T cells were both increased, while macrophages were reduced in kidneys of Apobec-1 KO animals. Overexpression of Apobec-1 in mouse proximal tubule cells protected against CP-induced cytotoxicity. These findings suggest that Apobec-1 mediates critical pro-survival responses to renal injury and increasing Apobec-1 expression could be an effective strategy to mitigate AKI.

## Introduction

There are at least 13.3 million cases of AKI globally and 1.7 million deaths per year^1^. AKI may lead to de novo or accelerated CKD and end stage kidney disease^2^. We have developed an animal model to study AKI, CKD, and the transition from AKI to CKD using the nephrotoxic drug cisplatin^3^. CP is an effective chemotherapy for many solid tumors. The predominant dose-limiting adverse effect of cisplatin is AKI, recurrent episodes of which result in CKD^4^. CP accumulates and causes injury to renal proximal tubule epithelial cells^5^. The animal model we developed mimics the development of CKD seen in patients undergoing treatment for cancer with cisplatin^6,7^.

We have shown that CP inhibits fatty acid oxidation^8^ and acute renal failure is accompanied by accumulation of free fatty acid, triglycerides and cholesterol in serum, urine, and kidney tissue^9^. Acute tubular necrosis is the hallmark of AKI^10^. Among various types of cell death, including the receptor-interacting protein kinase 1 (RIPK1)-regulated necroptosis^11^, ferroptosis^12^ was shown to contribute to the progression of AKI in animal models induced by CP^13^, IR injury^14^, oxalate nephropathy^14^, and folic acid^15^. Ferroptosis is characterized by lipid peroxide accumulation and controlled by ACSL4, an enzyme that converts long chain fatty acid to fatty acyl-CoA esters, without which ferroptosis cannot be executed^16^.

Apolipoprotein B [ApoB] is a large glycoprotein that serves an indispensable role in the assembly and secretion of lipids, including triglyceride and cholesterol of both dietary and endogenous origin, as well as the intravascular transport and receptor-mediated uptake and delivery of distinct classes of lipoproteins to distant organs^16^. ApoB circulates in two distinct forms, ApoB100 and ApoB48. ApoB48 arises following C-to-U deamination of a single cytidine base in the nuclear *ApoB* transcript, introducing a translational stop codon, by the action of Apobec-1^17,18^. Apobec-1 plays a crucial role in the regulation of lipid metabolism, as well as participating in stress responses to noxious stimuli, inflammation and repair of injured organs^17^. Recently it has been shown to play a role in the response to radiation injury in the small intestine not by its nucleotide editing role but by binding to and stabilizing Cyclooxygenase 2 mRNA and thereby increasing its protein levels and function^19^. Thus, the repertoire of APOBEC-1 effects on gene expression in injured organs occurs at transcriptional and post transcriptional levels. This broad range of activity and its demonstrated protective effect in injured organs, coupled with its widespread expression and activity (beyond liver and small intestine)^20^ make Apobec-1 an ideal candidate for study of genetic modifiers relevant to the injured kidney.

Therefore, we conducted this study to investigate the role of Apobec-1 in kidney injury using CP- and IR-induced kidney injury mouse models. Our results showed that APOBEC1 is crucial to the response of the kidney to these noxious stresses.

## Results

### *Apobec1* gene expression is induced in kidney after injury

**Figure 1:**
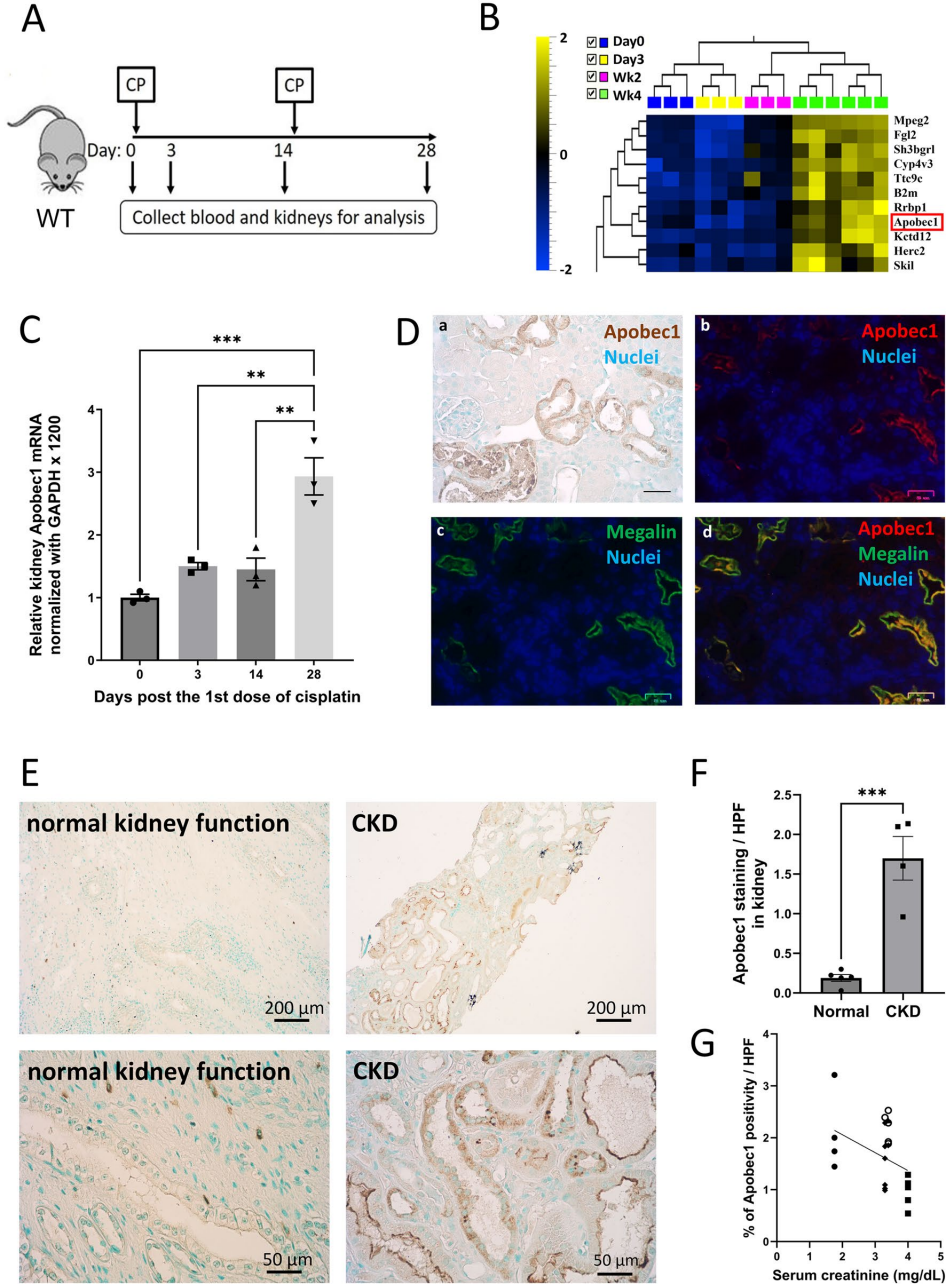
*Apobec 1* expression is induced in AKI and CKD. (**A**) Schematic representation of experimental design for mouse model of CKD. (**B)** Heat map of DNA microarray study showing a cluster of genes, include *Apobec1*, elevated at the transition from AKI to CKD. (**C**) Taqman assay of reverse transcribed real time PCR for *Apobec1* mRNA levels. The data represented as mean ± SE (n = 3), were subjected to unpaired t test (**p < 0.05, ***p < 0.001 in comparison to negative control). (**D**) Localization of APOBEC1 expression in mouse kidneys. Representative pictures of immunohistochemistry or immunofluorescence of kidneys from WT treated with cisplatin for 4d for APOBEC1 staining. Uninjured kidneys showed no expression. (**a**) immunohistochemistry of APOBEC1, nuclei were stained with methyl green; (**b**–**d**) immunofluorescence co-staining with APOBEC1 and megalin. Nuclei were stained with DAPI (blue). Scale bar is 50 µm. (**E**) Representative pictures of immunohistochemistry of human kidney biopsy from subjects with normal kidney function (left) or with CKD (right) for APOBEC1, nuclei were stained with methyl green. (**F**) Quantification of APOBEC1-positive area of human kidney biopsy per high power field (HPF) in CKD, unpaired t test, n = 4, ***p < 0.0005. (**G**) Correlation between serum creatinine and expression of APOBEC1 in 4 individual CKD patients’ kidney biopsy. Pearson correlation was used to measure the linear relationship between serum creatinine level and Apobec1 expression, R squared = 0.5.

To identify genes that are expressed during AKI and CKD development, we induced CKD in C57Bl/6J mice using two doses of CP (Fig. 1A). Mice were euthanized at days 3 and 14 after the first cisplatin dose, and 14 days after the second dose (28 days after the first dose). At each time point, we measured plasma creatinine and collected kidney tissue to confirm AKI and CKD development (data not shown). Microarray analysis revealed clusters of genes elevated at the transition of AKI to CKD (4 weeks) (Fig. 1B). *Apobec-1* was prominently expressed at the AKI-CKD transition and its increased expression mimics that found after injury to the small intestine and liver^21^. The increase in Apobec1 expression revealed by microarray analysis was confirmed using reverse transcription real time PCR (Fig. 1C), showing that apobec1 mRNA levels were increased 1.50-fold at Day 3 and 2.93-fold at Day 28 as compared to control mice receiving the isotonic saline vehicle alone.

*Apobec1* expression in mouse kidney was further examined by immunohistochemistry using 2 different antibodies against APOBEC1 (see “Methods”). Both antibodies labeled mouse kidneys in the same pattern: positive staining was seen in tubules (Fig. 1D image a). Preincubation with the APOBEC-1 peptide used to generate the antibody abolished the staining and no positive staining was observed in kidney from *Apobec-1* KO mice treated with cisplatin for 4d (Supplemental Fig. S1), indicating that the tubule staining is specific for the APOBEC-1 protein. Immunofluorescence co-staining for APOBEC-1 and MEGALIN, a marker for the proximal tubule, demonstrated APOBEC-1 expressed in proximal tubule (Fig. 1D, images b–d). IHC for APOBEC-1 expression was performed in human kidney sections of a patient with normal kidney function and kidney biopsy sections of 4 patients with CKD. Intense staining of APOBEC1 in tubules was observed in kidneys of all 4 subjects with CKD but not in kidney with normal kidney function (Fig. 1E and F). There is a trend (see Fig. 1G) that suggests the greater the reduction in renal function, the lower the expression of APOBEC1, even though there is an increase above control level of APOBEC1 expression (Fig. 1F) in CKD.

We interpret these results to indicate that AKI and CKD development is associated with an increase in APOBEC-1 expression in proximal tubules and that similar increased APOBEC-1 expression occurs in human diseased kidney tubules.

### Deletion of *Apobec1* leads to severe cisplatin-induced and ischemia reperfusion-induced AKI

**Figure 2:**
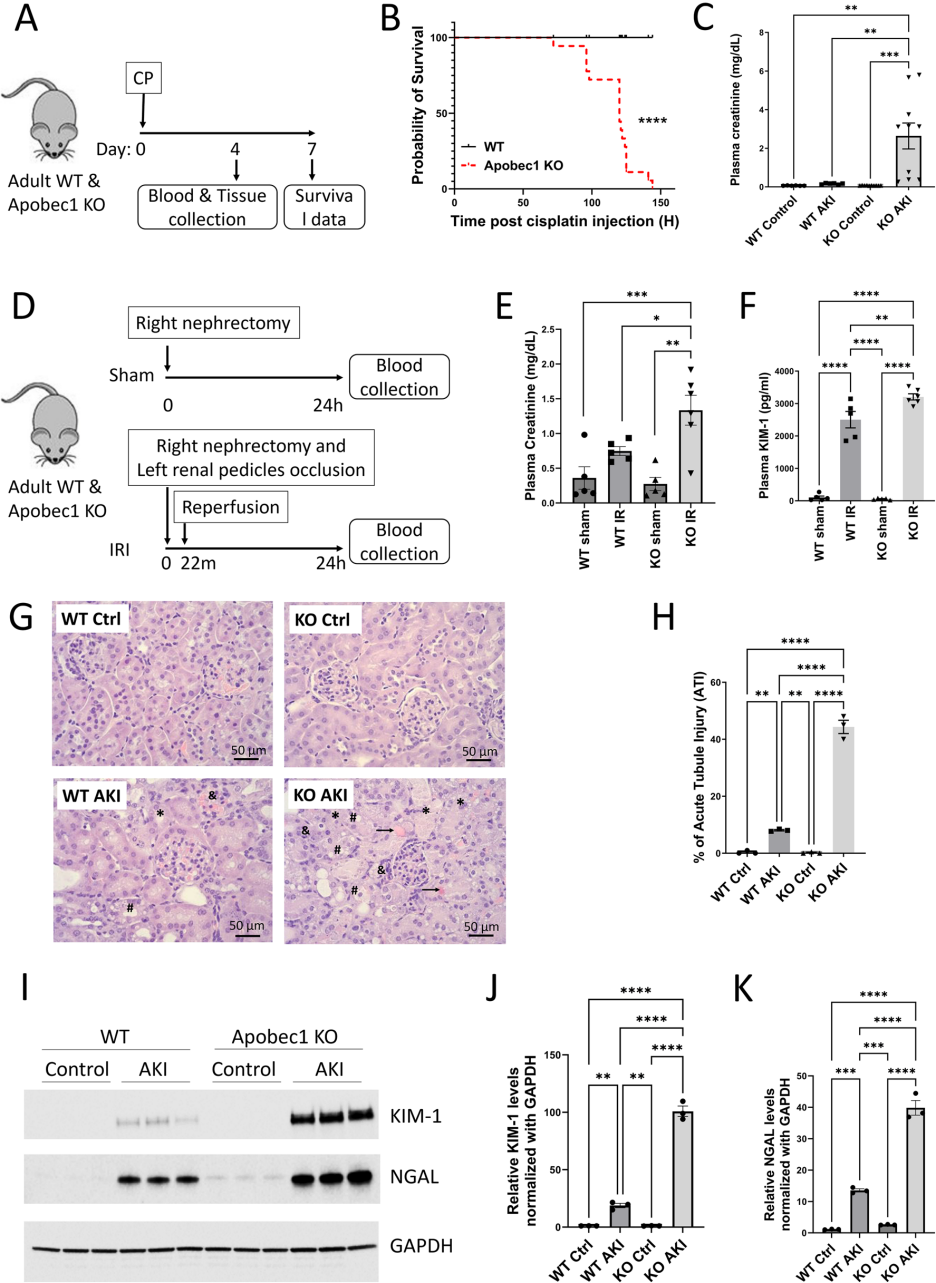
Deletion of *Apobec1* leads to severe cisplatin-induced and ischemia reperfusion-induced AKI. (**A**) Schematic representation of experimental design for mouse model of AKI. (**B**) Survival of WT versus KO mice after cisplatin treatments. Data from (**B**) were analyzed using Kaplan–Meier curves and a log-rank test, n = 18, ***p < 0.0001. (**C**) Plasma creatinine level at Day 4 post CP injection, one way ANOVA multiple comparison, n = 10, **p < 0.001, ***p < 0.0005 ****p < 0.0001. (**D**) Schematic representation of experimental design for mouse model of IR. (**E**) Plasma creatinine level 24 h after IR surgery, one way ANOVA multiple comparison, n = 5 in control and WT AKI groups and n = 6 in KO AKI group, *p < 0.05, **p < 0.01. (**F**) Plasma KIM-1 level 24 h after IR surgery, one way ANOVA multiple comparison, n = 5 in control and WT AKI groups and n = 6 in KO AKI group, **p < 0.01, ****p < 0.0001. (**G**) Representative figures of hematoxylin and eosin staining from different groups of CP-AKI mouse model. Renal damage indicators are represented: → : accumulation of protein casts in the renal tubules. & region: tubule swelling. *loss of brush border membrane. #cell debris detachment. (**H**) Acute kidney injury scores revealed by hematoxylin and eosin staining from different groups of CP-AKI mouse model. (**I**) Immunoblotting of kidney injury markers KIM-1 and NGAL in total kidney lysate from WT and KO mice treated with CP for 4 days, n = 3 from each group. GAPDH was used as a loading control. Note that cropped blots are displayed, uncropped blots are included in the supplemental information file. Quantification of KIM-1 (**J**) and NGAL (**K**) from immunobotting, one way ANOVA multiple comparison, n = 3, **p < 0.01, ***p < 0.0005, ****p < 0.0001.

To determine whether this increase of *Apobec-1* expression in AKI and CKD is central to the development of kidney injury, we compared the degree of kidney injury in WT and *Apobec-1* KO mice using 2 mouse models: CP- and IR-induced kidney injury. WT and *Apobec-1* KO mice were injected with 15 mg/kg cisplatin and kidney injury and repair were monitored over 7 days (Fig. 2A). Survival of the *Apobec-1* KO mice was significantly reduced starting at 4 days after cisplatin. Remarkably all *Apobec-1* KO mice died 6 days post CP, while WT animals all survived with this dose of cisplatin (Fig. 2B). *Apobec1* KO sustained more severe AKI as evidenced by Day 4 plasma creatinine levels: 2.64 ± 0.67 mg/dL for KOand 0.16 ± 0.015 for WT (Fig. 2C). Similarly, WT and *Apobec-1* KO mice were subjected to ischemia reperfusion (Fig. 2D), plasma creatinine values of KO were significantly higher (1.34 mg/dL ± 0.22) than WT (0.75 mg/dL ± 0.06) (Fig. 2E); plasma kidney injury molecule-1 (KIM-1) levels of KO mice were also significantly higher (3204 pg/ml ± 94.8)than WT (2502 pg/ml ± 253) (Fig. 2F). Hematoxylin and eosin staining of mouse kidneys showed more extensive injury with a 5.5-fold increase in acute tubule injury (ATI) in *Apobec-1* KO as compared with WT (Fig. 2G): 44.3 ± 2.3% ATI in KO AKI vs. 8.06 ± 0.23% ATI in WT AKI (Fig. 2H). The expression of injury markers KIM-1 and neutrophil gelatinase-associated lipocalin (NGAL) in kidneys was also augmented in *Apobec-1* KO kidneys compared to the WT mice (Fig. 2I-K). These data indicate that *Apobec-1* deletion is associated with severe tubule injury after cisplatin and ischemia reperfusion.

### *Apobec1* deletion is associated with inflammation in AKI

**Figure 3:**
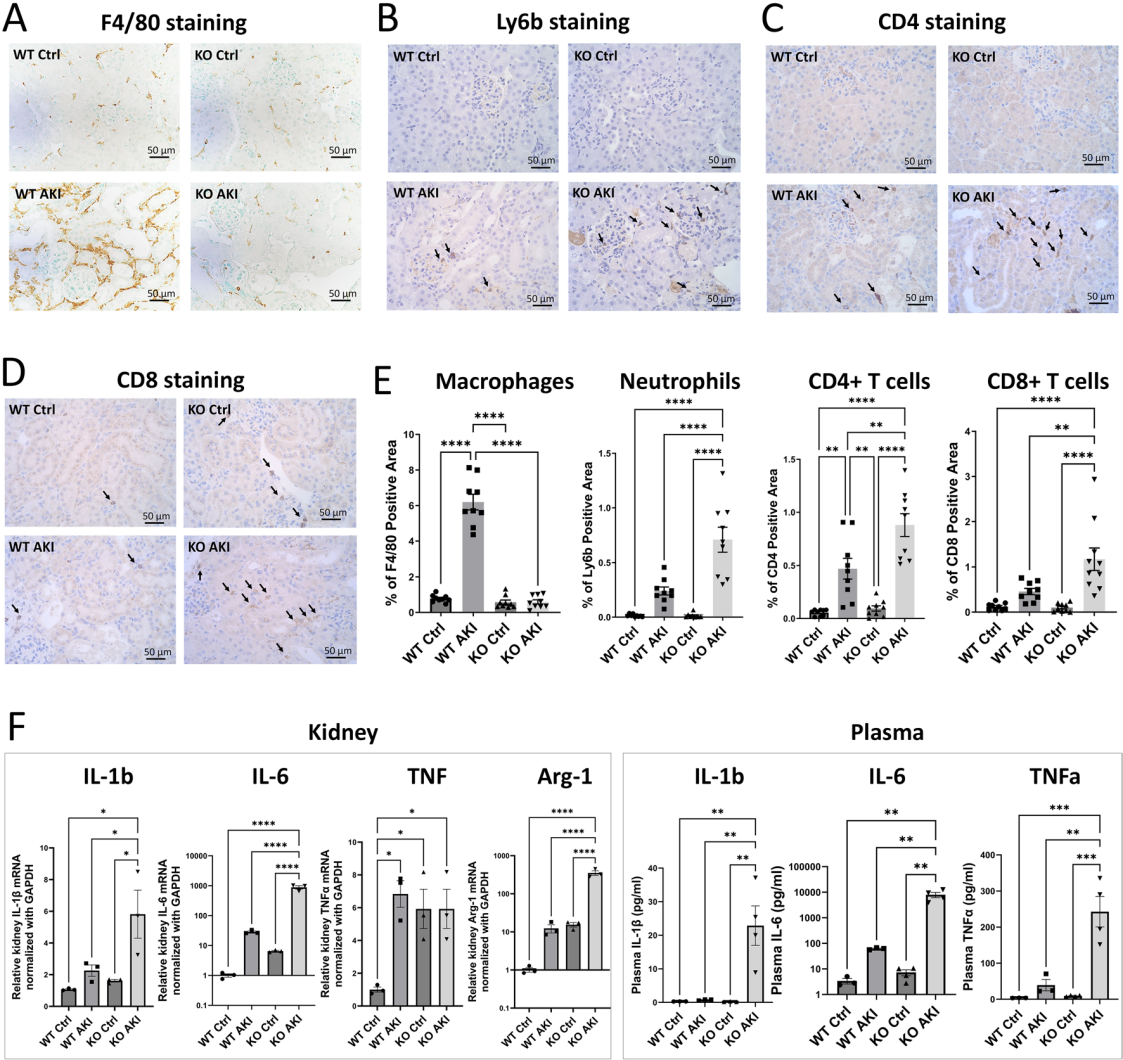
Decreased macrophage infiltration and elevated neutrophil and activated T cells in KO AKI kidneys. (**A**–**D**) Representative pictures of immunohistochemistry of formalin-fixed paraffin-embedded kidney tissues with F4/80 (**A**), anti-Ly6b (**B**), anti-CD4 (**C**), and anti-CD8 (**D**) from each group of mice in CP-AKI model. (**E**) Quantification of immune cell-positive area per high power field, one way ANOVA multiple comparison, n = 3, *p < 0.05, **p < 0.01, ****p < 0.0001. (**F**) Plasma and renal expression of IL-1β, IL-6, TNFα and renal expression of Arg-1 were assessed by one way ANOVA multiple comparison, n = 3, *p < 0.05, **p < 0.01, ***p < 0.005, ****p < 0.0001.

Inflammation is an important mediator of most forms of AKI. It was reported that *Apobec1* maintains the balance between the homeostatic and activated immune functions of macrophages and the absence of *apobec1* provokes a proinflammatory phenotype^22^, thus we performed staining to evaluate whether the absence of APOBEC-1 affected immune cells in CP-AKI (Fig. 3). F4/80 staining in WT mouse kidneys 4 days after cisplatin injection increased significantly: % of positive staining 0.781 ± 0.067 in control vs. 6.194 ± 0.440 in AKI. By contrast, no such induction was observed in KO kidneys after the induction of AKI: 0.578 ± 0.117% of positive staining in KO control vs. 0.592 ± 0.118 in KO AKI (Fig. 3A and E). This result indicates a defect of macrophage infiltration to kidney in *Apobec1* KO mice.

By contrast, we observed a significant increase in neutrophils and activated T cells in *Apobec1* KO AKI kidneys as compared with WT AKI kidneys. Neutrophils increased ~ threefold in KO versus WT animals after injury (Fig. 3B and E, 0.71 ± 0.11% of positive area per high power field in KO AKI vs. 0.24 ± 0.04% of that in WT AKI kidney), and CD4 + T cells increased ~ twofold in KO as compared to WT (Fig. 3C and E, 0.88 ± 0.11% of positive area per high power field in KO AKI kidney vs. 0.47 ± 0.01% positive area per high power field in WT AKI). A ~ threefold increase of CD8 + T cells was also seen (Fig. 3D and E, 1.17 ± 0.25% positive area per high power field in *Apobec1* KO AKI vs. 0.46 ± 0.07% positive area per high power field in WT AKI, *p* < 0.005, one way ANOVA). Note that there were areas of deep diffuse staining with Ly6b and CD4 antibodies, marked with black arrows in Fig. 3B and C, in KO AKI kidneys and these areas were excluded from quantification of positive area.

To confirm the heightened inflammatory response reflected by the increase in neutrophils and T cells that we observed in the Apobec1 KO AKI, we showed increased cytokines IL-1β, IL-6, TNFα in both kidney and plasma (Fig. 3F).

### Deletion of *Apobec*-*1* leads to altered gene expression in kidneys

**Figure 4:**
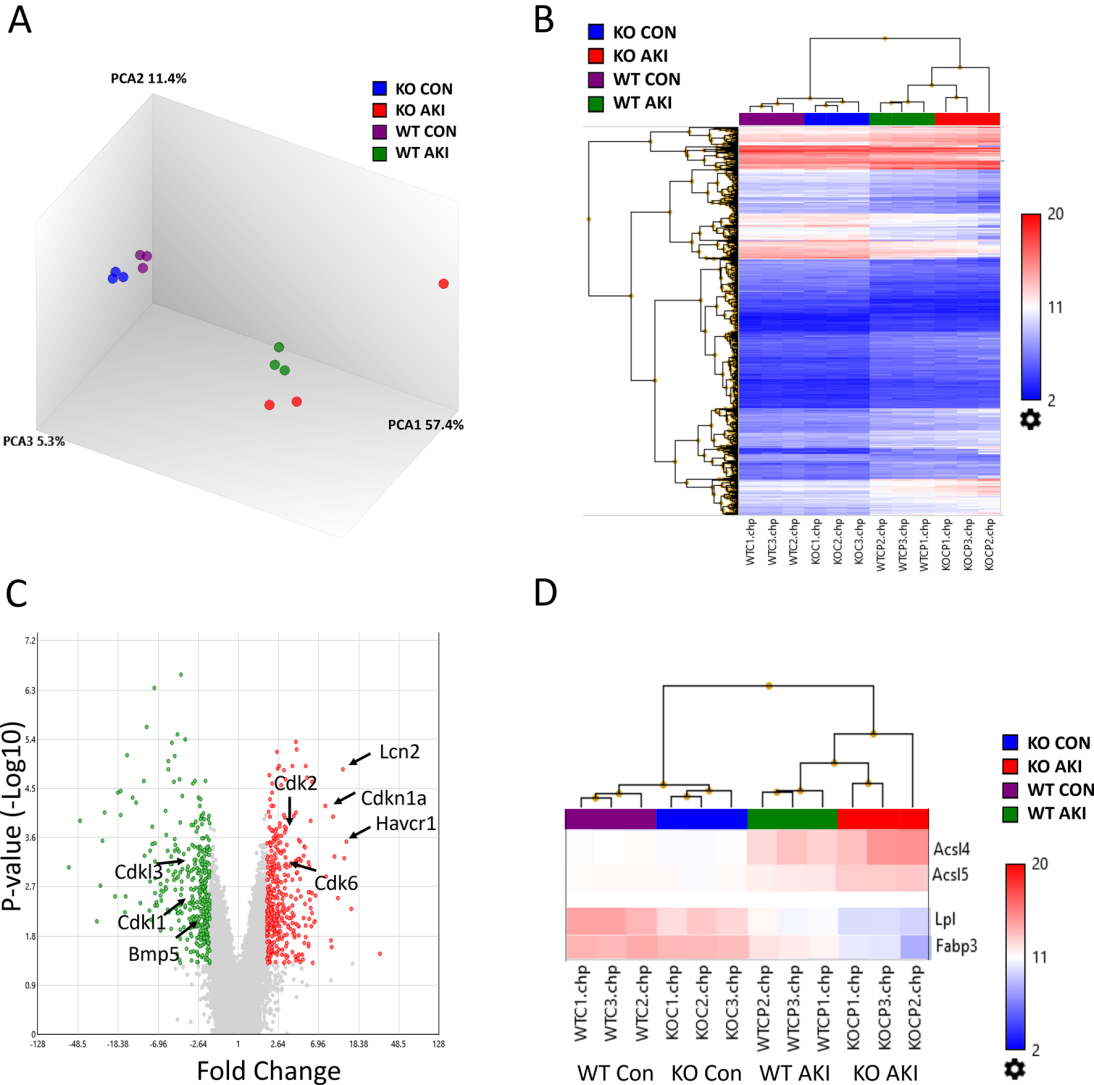
Changes in kidney transcriptional profile among Apobec1 KO and WT with or without AKI. (**A**) Principal component analysis of the multi-group analysis of variance comparison illustrating the significant variation between the gene expression profiles of the WT control (purple), WT AKI (green), KO control (blue) and KO AKI (red) animals. (**B**) Heat map and hierarchical clustering of transcripts, q = 0.001. (**C**) Volcano plot of Apobec1 KO AKI vs WT AKI, p < 0.001, fold change > 2 or -2. Green dots on left side represent genes downregulated in KO AKI and red dots on the right side represent genes upregulated in KO AKI as compared with WT AKI samples. (**D**) Heat map of the microarray study showing Acsl4, Acsl5, Lpl, and Fabp3.

We used DNA microarray technology to investigate the transcriptional profiling of genes modulated in kidneys of Apobec1 KO mice compared with WT, with or without AKI. Principal component analysis (Fig. 4A) and unsupervised hierarchical clustering analysis (Fig. 4B) demonstrated that each treatment group of samples clusters separately. The variability of the array data is unexplained. Comparison of Apobec1 KO and WT control (P < 0.01, fold Change: > 2 or < 2) identified 202 differentially expressed genes, while there were 756 differentially expressed genes between the KO and WT AKI samples. When analyzed in a database of Ingenuity Pathway Analysis, these differentially expressed genes were significantly associated with oxidative stress and damage, cell cycle control and cell death, fatty acid biosynthesis and oxidation, inflammatory response, and PPAR signaling (Table 1, Fig. 4C and D). Cisplatin administration resulted in more oxidative stress and damage in Apobec1 KO kidney. Similar to Western blot analysis (Fig. 2I), kidney injury markers Havcr1, also named kidney injury molecule–1 (KIM-1) and Lcn2, known as neutrophil gelatinase-associated lipocalin (NGAL), are highly expressed in KO kidney (Fig. 4C). So did the cell cycle arrest gene cyclin dependent kinase inhibitor 1A (Cdkn1a; p21). By contrast, cyclin dependent kinase like protein 1 and 3 (Cdkl1 and Cdkl3), which are known to promote cell proliferation, were downregulated (Fig. 4C). Reno-protective molecule Bone morphogenetic protein 5 (Bmp5) was also downregulated in KO AKI (Fig. 4C). Upregulation of CD36, Acsl4, and Acsl5 (Fig. 4D and E) in KO AKI was of particular interest given their known role in fatty acid transport and ferroptosis.

**Table 1:** Analysis of Apobec1-specific genes involved in CP-AKI by DNA microarray.

Function	Significance	Focus molecules	Molecules in network
Oxidative stress and redox pathway	4.86	17	CD44, Gls, Ggt1, Slco1a1, Slco2a1, Pgd, Gpx4, Cbs, Grxcr1, Gclc, Gsta1, Gsta2, Gstm3, Gstk1, Txnrd1, Txnrd3, Sod1
Oxidative damage response	3.41	9	Cdkn1a, Gadd45a, Pcna, Apaf1, Bak1, Bcl1, C2, Map2k6, Traf1
Ferroptosis			Acsl4,
Apoptosis	3.28	13	Tnfrsf10b, Tnfrsf1a, Traf1, Birc3, Ikbkg, Nfkbia, Bak1, Bcl2, Bcl2l1, Trp53, Casp6, Casp8, Apaf1
Fatty acid biosynthesis	2.96	6	Echdc1, Pecr, Scd1, Acsl4, Acsl5, Acss2
Fatty acid omega-oxidation	2.27	3	Cyp1a2, Cyp2e1, Aldh1a1
Fatty acid beta-oxidation	1.89	6	Lpl, Gcdh, Acat1, Acss2, Acsl4, Acsl5
Inflammatory response	1.06	4	Il5ra, Tnfrsf1a, Fn1, Lamc1
Toll-like receptor signaling	0.9	9	Tlr4, Tlr8, Ly96, Ifnar1, Casp8, Ikbkg, Map2k6, Nfkb2, Nfkbia
Cell Cycle control	0.78	6	Cdkn1a, Cdk2, Cdk6, Trp53, Gadd45a, Pcna
PPAR signaling	0.64	7	Cd36, Fabp3, Scd1, Dbi, Lpl, Acsl4, Acsl5
Mapk signaling	0.62	13	Egf, Map4k4, Map2k6, Gna12, Pak2, Casp6, Casp8, Ikbkg, Gstm3, Gsta1, Gsta2, Cyp2e1, Trp53

Top functions associated with changes in kidney transcriptional profile between Apobec1 KO AKI and WT AKI. Top diseases and functions (P < 0.01, Fold Change: > 2 or < 2) identified from a general (non–organ specific) database for 1253 transcripts identified by ANOVA. Significance was calculated from the p-score (p-score =  − log [P value]) and focus molecules are the number of molecules that appear in network and in the dataset.

### Deletion of *Apobec*-*1* leads to lipid accumulation in AKI-kidneys

**Figure 5:**
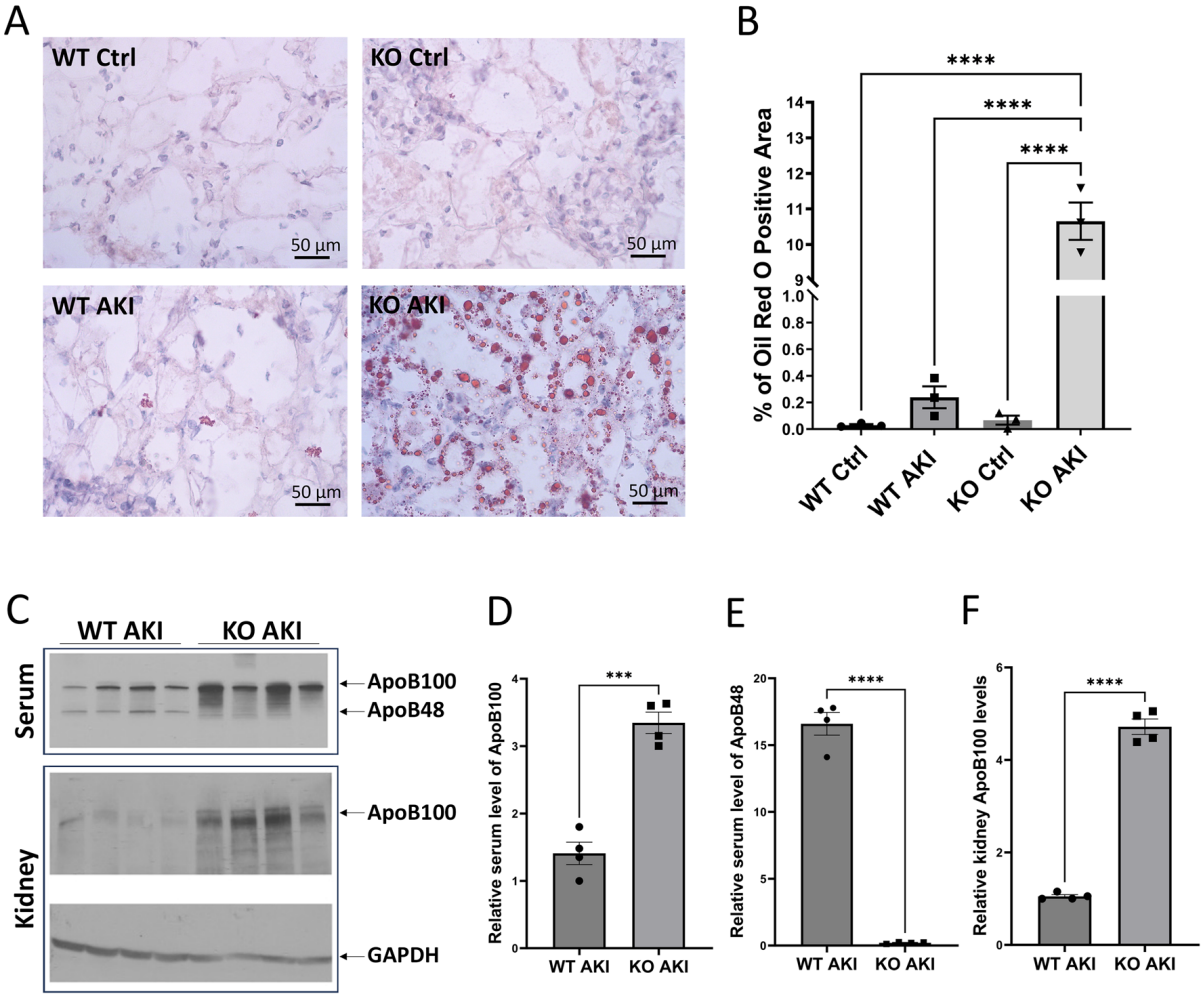
Increased lipid accumulation and ApoB in Apobec1 KO of CP-AKI. (**A**) Representative pictures of Oil Red O staining of frozen kidney Sects. (40 × magnification). (**B**) Quantification of the Oil Red O-positive area per high power field, one way ANOVA multiple comparison, n = 3, ****p < 0.0001. (**C**) A representative immunoblot for ApoB in serum (upper panel), in kidney (middle panel) and GAPDH in kidney (lower panel). Note that cropped ApoB and GAPDH blots are displayed, uncropped blots are included in the supplemental information file. (**D**–**F**) Quantification of serum ApoB100, ApoB48, and kidney ApoB100 detected by immunoblotting, respectively, one way ANOVA multiple comparison, n = 4, *p < 0.05, **p < 0.01, ****p < 0.0001.

Recent evidence suggests that AKI is accompanied by significant metabolic abnormalities, including alterations in lipid metabolism^23^. Since APOBEC1 edits ApoB100 to the shorter form ApoB48, which is the main apolipoprotein of chylomicrons and low-density lipoprotein, and is a key component in lipid metabolism, we performed oil red O staining to localize triglyceride and lipids in the injured kidney. We found extensive lipid deposits in *Apobec-1* KO AKI kidneys (Fig. 5A). There was a 45-fold increase in Oil Red O staining in KO AKI kidneys as compared to WT AKI (10.7 ± 0.52 in KO AKI vs. 0.24 ± 0.08% in WT AKI kidney) (Fig. 5B). Immunoblot analysis for APOB (Fig. 5C) in WT and APOBEC1 KO AKI kidneys shows that the ApoB48 serum levels increase significantly during AKI in WT while no ApoB48 was detected in Apobec1 KO serum. By contrast, ApoB100 increased significantly in the KO to a great extent than was found in WT (Fig. 5C–F). Cisplatin-induced AKI increased APOB100 in WTkidneys (1.01 ± 0.4 in WT control vs. 75.9 ± 26.4 in WT AKI). However, while the *Apobec-1* KO kidney at baseline displayed an elevated APOB100 compared to WT at baseline, the KO AKI kidney expressed nearly tenfold more APOB as compared to WT AKI: 515.1 ± 93.42 vs. 75.91 ± 26.4 in WT AKI (Fig. S2). These data suggest that the absence of APOBEC-1 has significant effects on baseline *ApoB* expression in the kidney that was enhanced during renal stress induced by cisplatin.

### Cisplatin administration results in elevated regulated necrosis proteins in *Apobec1* KO kidneys

**Figure 6:**
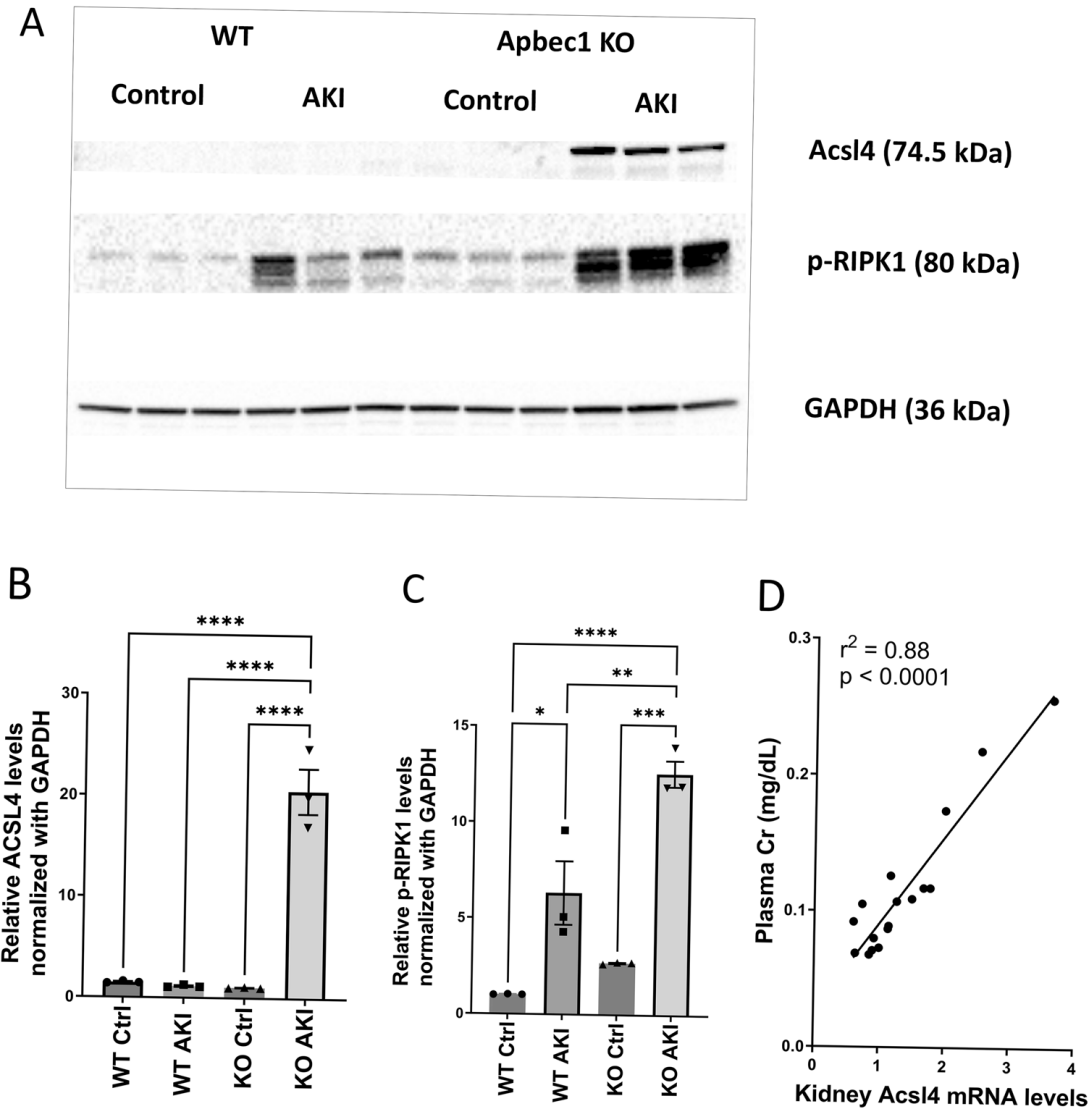
Expression of Acsl4, phosphorylation of RIPK1 as marker of ferroptosis, lipotoxicity, and regulated necrosis, respectively, after cisplatin administration. (**A**) Immunoblotting analysis of protein isolated from kidneys at day 4 after cisplatin administration. Three individual mice per treatment group were analyzed on the blots. Note that cropped blots of Acsl4 and phosphorylated RIPK1 (p-RIPK1) are displayed, uncropped blots are included in the supplemental information file. (**B** and **C**) Quantifications of immunoblots shown in (**A**), GAPDH was used as loading control. Differences were analyzed for significance using one way ANOVA corrected for multiple comparisons. *p < 0.05; **p < 0.001, ***p < 0.0005, ****p < 0.0001. (**D**) Correlations between plasma creatinine levels and expression levels of Acsl4.

The observation that lipid accumulation was markedly enhanced in the KO animals undergoing AKI led us to examine levels of acyl-CoA synthetase long-chain family member 4 (ACSL4), which is an essential pro-ferroptotic gene^24^, using immunoblotting. As shown in Fig. 6A and B, ACSL4 was markedly increased in KO AKI. On the other hand, no such increase in ACSL4 occurred in injured WT kidneys. Receptor-interacting protein kinase 1 (RIPK1), a marker for regulated necrosis, was examined by immunoblot analysis as well. The phosphorylated-RIPK1 showed significantly increased expressions in *Apobec-1* KO AKI (Fig. 6A and C): 12.59 ± 0.689 relative intensity of KO AKI kidneys vs. 6.328 ± 1.649 of WT AKI. Interestingly, increased ACSL4 expression in kidney bore a direct relationship with increasing plasma creatinine (Fig. 6D), suggesting that ferroptosis is the major cause of kidney failure in CP-AKI.

### Overexpression of *Apobec1* in TKPTS protected cells from cisplatin-induced cytotoxicity

**Figure 7:**
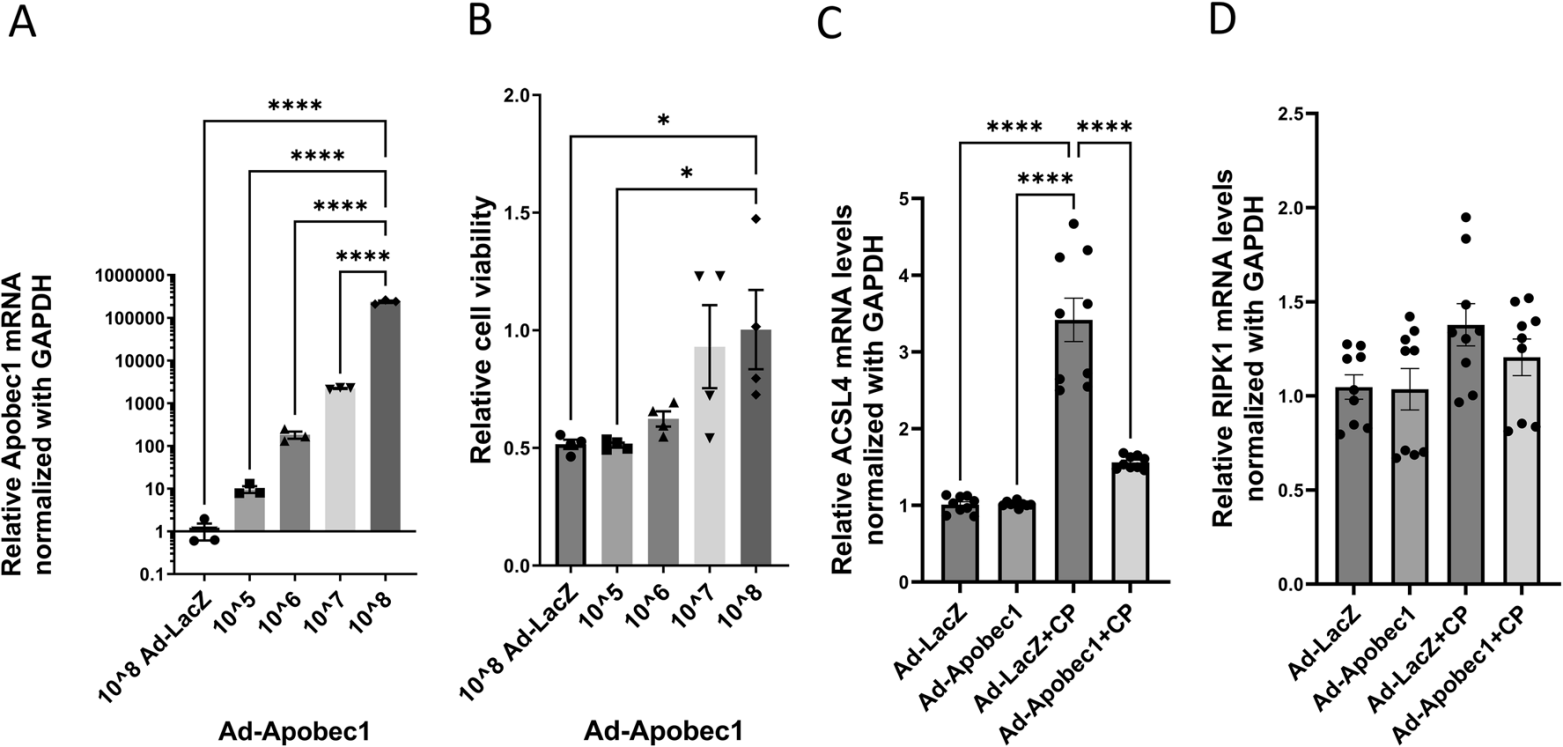
Overexpression of *apobec1* protected proximal tubule cells from CP-induced cytotoxicity. Mouse proximal tubular cells TKPTS were transduced with adenovirus overexpression lacZ (Ad-LacZ) or *Apobec1* (Ad-Apobec1) for 16 h, followed by 24-h treatment with or without 25 µM cisplatin. (**A**) Relative *Apobec1* mRNA levels in TKPTS cells transduced with Ad-Apobec1 or Ad-LacZ 40 h after transduction. n = 4, **p < 0.01, ****p < 0.0001. (**B**) A representative WST-1 assay: the relative cell viability is expressed as mean ± SEM, n = 4, differences were analyzed for significance using one way ANOVA corrected for multiple comparisons. *p < 0.05. (**C** and **D**) Taqman assays of reverse transcribed real time PCR for Acsl4 and RIPK1 mRNA levels. The data represented as mean ± SE (n = 9), differences were analyzed for significance using one way ANOVA corrected for multiple comparisons, ****p < 0.0001.

The effect of APOBEC-1 overexpression on CP-induced cytotoxicity was further examined in vitro using mouse kidney tubule cells line TKPTS. The advantage of this approach is that these cells do not express Apobec1. TKPTS cells were transduced with control adenovirus Ad-LacZ or rat Apobec1 over-expressing virus (Ad-Apobec1) at different titers for 16 h and followed 24 h after CP exposure. The rat *Apobec1* mRNA levels increased as the virus titer increased (Fig. 7A). In parallel wells, cell viability was accessed with WST-1 assay. Increased expression of *Apobec1* (Fig. 7A) led to increased cell viability (Fig. 7B and Fig. S3), indicating *Apobec1* protected renal tubule cells from CP-induced cytotoxicity (Fig. 7A). The mouse Acsl4 mRNA level was elevated to more than threefold by CP in control cells (Fig. 7C). By contrast, no significant increase of Acsl4 mRNA by CP was observed in cells overexpressing Apobec1 (Fig. 7C), suggesting that A prominent form of cell death induced by cisplatin was ferroptosis (Fig. 7D). Although we could not detect an increase in RIPK1 mRNA, other forms of regulated necrosis may also be present.

## Discussion

Our findings reveal a remarkably increased sensitivity of the kidney to cisplatin in the absence of *Apobec-1*. For that reason, we focus on AKI before embarking on developing a model that would allow us to conditionally manipulate the Apbec-1 gene in the kidney. We are not aware of any single gene KO that has such a devastating effect on the kidney after exposure to cisplatin. A usually well tolerated dose of cisplatin^6^ caused extensive damage to the kidney and accompanying severe renal failure without recovery and ending in the death of the animal. It is possible that some of these animals may have had other organ damage that participated in their death. We focus on its renal effect in this study. More severe ischemia/reperfusion injury was also revealed in the knockout animals suggesting that the sensitivity extends to many forms of AKI. Increasing the expression of *Apobec-1* gene by transfection in the TKPTS cells also revealed its protective effects and demonstrates that the protection is cell autonomous. Perhaps most importantly for the translational aspect of the observation is that human CKD kidney specimens had increased expression of the APOBEC-1 protein in proximal tubules suggesting that *Apobec-1* expression may be necessary for limiting injury to the kidney in human disease as well. This is also reflected by the observation that more renal injury is accompanied by a less vigorous increase in APOBEC1.

The role of inflammation in AKI has been increasingly appreciated with involvement of leukocytes, adhesion molecules, chemokines, and cytokines^25^. Here we observed sustained increase in neutrophils and activated T cells (CD4 + and CD8 + T cells) as well as increased proinflammatory cytokines in the kidneys and plasma of AKI *Apobec1* KO mice. It is known that cisplatin administration causes an increase in kidney neutrophil content^26^, and anti-inflammatory agents, such as anti-TNF- antibodies, IL-10, and anti-intercellular adhesion molecule-1 antibodies, reduced renal neutrophil infiltration^26–29^. However, depletion of neutrophil did not improve renal function and tubular necrosis, suggesting that infiltrating neutrophils are not essential for cisplatin-induced renal injury^30^ and may be a reflection of the severity of injury rather than its cause^31^. T cell-deficient mice have greater renal dysfunction, reduced survival, and increased infiltration of neutrophils and macrophages into the kidney in CP-AKI^32^, suggesting that both CD4 and CD8 T cells play role in cisplatin toxicity^32^. As such, the sustained accumulation of neutrophil, CD4 + and CD8 + T cells in the KO AKI kidneys indicate a greater kidney injury.

Although there was no difference in number of macrophages at baseline between WT and *Apobec1* KO, *Apobec1* KO mice attenuated CP-induced macrophage infiltration, suggesting that *Apobec1* is necessary for bone marrow derived macrophages and monocytes to home to injury sites. Our observations fit well with what is known about the enhanced inflammation and decreased phagocytosis and trans endothelial migration demonstrated by macrophages collected from *Apobec1* KO mice^22,33^. Whether these changes in immune cell function is primarily responsible for the sensitivity of the KO animals to cisplatin is unknown at the present time. In future work, we plan to measure daily macrophage infiltration to observe the full extent of the macrophage response to renal injury in the absence of Apobec1 gene and use targeted approaches to demonstrate conclusively the role of macrophage infiltration and polarization play in nephrotoxicity.

The precise identity of the pathway(s) responsible for this greater sensitivity was not addressed in this study, yet several observations merit further exploration. Regulated necrosis was enhanced in the absence of the *Apobec-1* gene. This was particularly true of the ferroptotic pathway given the prominent activation of the master regulator of the pathway, ASCL4, in the KO kidney. All of the elements of the pathway were present including an increase in triglyceride content as revealed by the increased Oil Red O staining demonstrated in the KO after cisplatin injection. Thus, in the background of the well-known increase in triglyceride and fatty acid content of the proximal tubule in animals given cisplatin^8^ as well as the increased oxidant stress that accompanies cisplatin-induced AKI^34^, the enhanced expression of *acsl4* would be expected to have a devastating effect on the kidney as it enriches cellular membranes with long polyunsaturated ω6 fatty acids. These long-chain fatty acids activate AMPKβ1-containing isoforms to increase fatty acid oxidation^35^. The effect of APOBEC-1 to generate the shortened version of ApoB48, which is well known to be the favored pathway for intestinal cells to dispose of ingested and reabsorbed triglycerides, suggests that it may be necessary for the injured kidney to use a similar pathway to limit the accumulation of these potentially damaging lipids. Formal analysis of this hypothesis however awaits future study.

Yet the repertoire of the gene changes induced by the absence of *Apobec1* goes far beyond its effect on lipid disposal. The enhanced increased susceptibility of intestinal cells undergoing radiation injury depends on the effect of apobec1 to enhance the translation of the cylooxygenase-2 mRNA^19^, the accelerated aging of *Apobec-1* KO mice depends on the enhanced microglial expression of inflammatory genes^22^, and the regeneration of the liver depends on the expression of the *Apobec1* Complement Factor^36^ and its effect on the stability of the IL-6 mRNA^37^ suggesting the complex repertoire and potential targets of *Apobec-1*.

**Figure 8:**
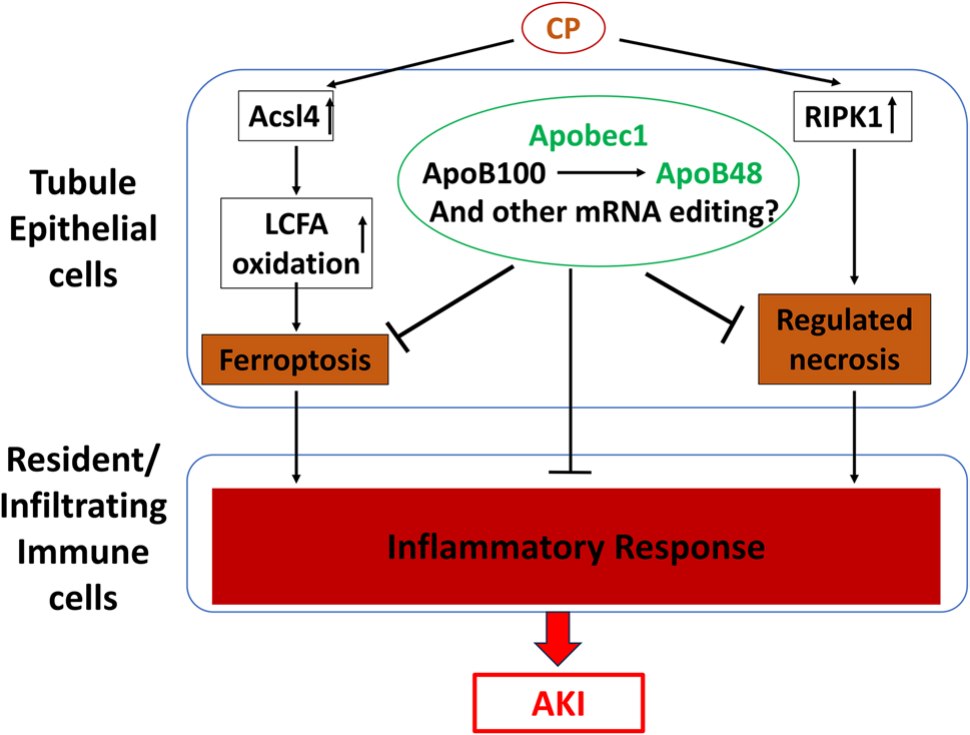
Proposed mechanism mediating the protective action of APOBEC1 against cisplatin-induced AKI. APOBEC1 is crucial to mitigate cisplatin-induced renal injury possibly through inhibiting lipotoxicity, ferroptosis, regulated necrosis, and the inflammatory response to cisplatin.

In summary, we demonstrate that *Apobec-1* is crucial to survive from CP-induced nephrotoxicity. The absence of *Apobec1* revealed a remarkable sensitivity to cisplatin at a dose that is normally well tolerated. Apobec1 affects mRNA and protein expression of many genes, and this function is crucial to mitigate cisplatin- or IR-induced AKI. The absence of Apobec1 lead to an increase of Acsl4 expression. Although Acsl4 is not known to be edited by Apobec1, it is possible that Acsl4 is affected indirectly. We indicate this possibility by the arrows connecting them in Fig. 8.

Upregulation of *apobec1* is a critical pro-survival response to renal injury and increasing its activity could be an effective strategy to reduce or prevent CP-induced AKI. This possibility can be tested in vivo by delivery of Apobec-1 to the kidney. Further studies to identify the precise pathway (s) responsible for the salutary effect of apobec1 will be necessary to design effective strategies to mitigate injury after nephrotoxic and ischemic stress to the kidney.

## Methods

### Animal models

Mice were maintained at Veterans Affair Medical Center (VAMC), West Haven, CT. All experimental procedures were conducted according to the guidelines and regulations for animal care and use by the Institutional Animal Care and Use Committee of the VAMC and the authors complied with the ARRIVE guidelines. Mice were kept under a 12-h day/night cycle with food and water provided ad libitum. All experiments were repeated on at least two separate occasions. We used the ARRIVE1 reporting guidelines to report the animal studies.

#### Murine model of cisplatin-induced chronic kidney disease (CP-CKD)

WT C57BL/6 J mice were purchased from Jackson Laboratory. CP was purchased from Sigma-Aldrich (Saint Louis, MO, USA) and freshly prepared in sterile 0.9% sodium chloride (saline) at 0.875 mg/ml before given to animals. Adult mice (6–8 months) were fasted for 18 h and administered 2 doses of cisplatin at 15 mg/kg subcutaneously, 2 weeks apart. The animals were sacrificed, blood and kidneys were collected at designated time points.

#### Murine model of cisplatin-induced acute kidney injury (CP-AKI)

Homozygous *Apobec-1* KO mice were generated as previously described^18^. The KO mice have been generated by backcrossing with C57BL6J for 10 generations to preserve the C57BL6J background. The disruption of Apobec1 gene in the KO mice was confirmed by both genotyping and mRNA levels. Adult WT and *Apobec1* KO mice were administered a single dose of CP (15 mg/kg) subcutaneously. Control mice were administered saline. Four days post CP, the animals were sacrificed, blood and kidneys were collected. No significant difference in the degree of injury was observed between male and female littermates.

#### Murine model of I/R injury

Male adult mice of WT and *Apobec-1* KO were anesthetized and subjected to IR injury. Male mice were used for IR due to substantial variability in susceptibility to IR between male and female mice. IR procedures were performed as a service provided by the George O'Brien Kidney Center at Yale University. Briefly the left renal pedicle was clamped for 22 min using a nontraumatic vasculature clamp (Fine Science Tools), whereas the right renal pedicle and ureter were ligated with two sutures and the right kidney removed. Reperfusion after release was considered successful if the kidney rapidly recovered its original color. Mice were kept at 37 °C using a warming pad during the procedure and closely monitored during recovery. Mice were given 0.5 mL of normal saline intraperitoneally to prevent dehydration. Blood and kidneys were obtained 24 h after reperfusion.

### Measurement of plasma creatinine, blood urea nitrogen (BUN), kidney injury molecule-1 (KIM-1), IL-1β, IL-6, and TNFα

Plasma creatinine levels were measured by capillary electrophoresis at the O’Brien Kidney Center at UT-Southwestern and BUN levels were determined using a Stanbio™ urea nitrogen direct kit (Thermo Fisher Scientific, Waltham, MA, USA) according to manufacturer’s instructions. Plasma KIM-1 levels were measured using the mouse TIM-1/KIM-1/HAVCR Quantikine ELISA Kit (R&D Systems, Minneapolis, MN, USA) according to manufacturer’s instructions. Plasma IL-1β, IL-6, and TNFα were examined using a mouse multiplex proinflammatory panel 1 kit (Cat # K15048D, Meso Scale Discovery, Rockville, MD, USA).

### Histology, immunohistochemistry, and oil red O studies

Human kidney biopsy from CKD patients were obtained from the previously described Yale biopsy cohort^38–40^. All methods were performed in accordance with the relevant guidelines and regulations by Yale University Human Subjects Committee for social, behavioral and educational research. Briefly, we enrolled consecutive patients undergoing a kidney biopsy at two Yale-affiliated sites, Yale New Haven Hospital and Saint Raphael’s Hospital. Both hospitals are in New Haven, CT. The study was approved by the Yale Human Investigation Committee under approval number 11110009286. All participants provided written informed consent. This sub-study includes participants enrolled between August 2015 and December 2016. For this sub-study we selected participants with baseline (pre-biopsy) estimated glomerular filtration rate (eGFR) < 30 ml/min/1.73m^2^, age > 60 years, and with histological diagnosis of arterionephrosclerosis. Characteristics of these participants are presented in Supplemental Table S1.

Slices of kidney tissue were embedded in paraffin and stained with Hematoxylin and Eosin for routine histology. For immunohistologic studies, sections were deparaffinized, rehydrated, transferred into citrate buffer, and heated to boil for 1 min. Sections were blocked with 3% hydrogen peroxide for 10 min and 5% BSA-TBST for 1 h, incubated with the primary antibody overnight at 4 °C. The following antibodies were used: anti-APOBEC-1 Catlogue # PA5-11429, Invitrogen, Carlsbad, CA, USA), anti-megalin (Catlogue # ab76969, abcam, Waltham, MA, USA), anti-Ly6b (Catlogue # MCA771G, BioRad, Hercules, CA, USA); anti-CD4, anti-CD8, and F4/80 (Catalogue # 25229, 98941, and 70076, respectively, Cell Signaling Technologies, Danvers, MA, USA). Signals of all primary antibodies were detected using SignalStain® Boost IHC Detection Reagent (Cell Signaling Technologies, Danvers, MA, USA). An APOBEC-1 peptide Ac-DVFYDPRELRKEAC-NH2 was synthesized by Lifetein, LLC (Somerset, NJ, USA) and used to test the specificity of APOBEC-1 antibodies. For staining triglyceride and lipid, kidneys were embedded in OCT, snap frozen, cryo-sectioned and stained for Oil Red O at Yale Histology Research Laboratory.

A renal pathologist, masked to the identity of the study animal, analyzed multiple sections from each kidney and the renal tissue sections evaluated for injury from hematoxylin and eosin staining. Tissue sections were scored using a square grid technique. Small squares of a 10 × 10-integrated grid, falling on areas with morphologic features of overt necrosis (sloughing of cells, brush border loss, blebbing of cytoplasm) were counted. Ten independent fields were counted per kidney (1000 squares per kidney), and the percentage of lesion area was calculated as percentage of total squares counted. Immunohistochemical positivity for one of the above tested markers were evaluated quantified using ImageJ software (National Institute of Health).

### Measurement of mRNA levels

RNA was extracted using RNeasy kit (QIAGEN, Germantown, MD, USA) according to the manufacturer’s instructions. RNA was reverse transcribed into cDNA using QuantiTect Reverse Transcription Kit (QIAGEN, Germantown, MD, USA) according to the manufacturer’s protocol using 1 mg RNA in a total of 20 µl reaction. Relative expression levels of various genes were assessed by quantitative PCR. The mRNA levels of *Apobec-1*, *ApoB*, *Acsl4*, and *Gapdh* were assessed using the TaqMan Gene Expression real-time PCR assays (Applied Biosystems, Foster City, CA, USA, Catalogue # Mm01184109_m1, Mm01545150_m1, Mm00490331_m1, and Mm99999915_g1, respectively). The results were expressed as the threshold cycle (Ct). The relative quantification of the target transcripts normalized to the endogenous control *Gapdh* was determined by the comparative Ct method (ΔCt) and the 2-ΔΔCt method was used to analyze the relative changes in gene expression between the tested cell lines according to the manufacturer’s protocol (User Bulletin No. 2, Applied Biosystems, Foster City, CA, USA).

### Microarray analysis

Microarray analyses were performed commercially using either OneArray® (Phalanx Biotech Group, San Diego, CA, USA) with RNA samples isolated from control, 3 day/1 dose, 2 week/1 dose, and 4 week/2 dose mice as previously described^7^ or using Clariom™ S assay (Catalogue # 902930, Thermo Fisher Scientific, Foster City, CA) with kidney RNA isolated from WT mice or Apobec1 KO mice treated with saline or CP 3 days after a 15 mg/kg dose. Data were analyzed as described previously^7^.

### Immunoblotting

Protein samples were prepared from mouse kidneys and mouse proximal tubule TKPTS cell line. Fifty µg of total proteins were separated on 4–15% stain-free premade TGX gels (BioRad, Hercules, CA, USA) and immunoblotted as described previously^7^. Images of the membranes after transfer were taken using the Criterion Stain-Free Imager (BioRad, Hercules, CA, USA). Blots were then incubated for overnight at 4 °C with primary antibodies specific to ApoB (prepared from hybridoma in house), GAPDH and phospho-RIPK1 (Caltalogue # 2118 and 53,286, respectively, Cell Signaling Technologies, Danvers, MA, USA), KIM-1 (Catalogue # AF1817, R&D Systems, Minneapolis, MN, USA), ACSL4 (Catalogue # SAB2100035, Millipore Sigma, Burlington, MA, USA). All antibodies were diluted 1:1000 (except KIM1, 1:400) in 5% bovine serum albumin. The blots were washed with TBST and incubated with horseradish peroxidase conjugated anti-rabbit (Catalogue# 7074, for p-RIPK1, ApoB, ACSL4, Cell Signaling Technologies, Danvers, MA, USA), or anti-goat (Catalogue # AP180P, for KIM1, Sigma, St Saint Louis, MO, USA) IgG diluted 1:2000 in 5% nonfat milk. Proteins were detected using a chemiluminescent substrate (SuperSignal® West Femto; Thermo Scientific, Waltham, MA, USA). Protein bands were quantitated using ImageJ software (U. S. National Institutes of Health, Bethesda, MD, USA). The intensity of each of the bands were normalized to the intensity of the GAPDH band.

### Overexpression of Apobec-1 in TKPTS

Mouse proximal tubule TKPTS cells were purchased (American Tissue and Cell Collection, Manassas, VA, USA) and maintained in DMEM: F12 supplemented with 7% fetal bovine serum and 6 µg/ml insulin (Sigma, St Saint Louis, MO, USA). Adenovirus expressing rat *Apobec1* cDNA or LacZ (as a negative control) were prepared as previously described^41^. Cells were transduced in OptiMEM-I medium (Thermo Fisher Scientific, Waltham, MA, USA) with Ad-Apobec-1 at amount indicated for 16 h, medium was replaced with growth medium and returned to culture. Cells were treated with or without 25uM CP for indicated time periods. Cells were assayed for viability using WST-1 reagent (Catalogue # 5015944001, Millipore Sigma, Burlington, MA, USA), or collected for RNA preparation.

### Statistical analyses

Statistical analyses were performed using GraphPad Prism 7.01 (GraphPad Software, San Diego, CA). The Wilcoxon rank test and the Mann–Whitney test were used for paired and unpaired data, respectively. When appropriate, unpaired *t* test and nonparametric repeated measures ANOVA (Friedman test) was used to evaluate statistical significance. When the Friedman test revealed statistical significance, Dunn’s test was used for pairwise comparisons. A Kaplan–Meier survival analysis was carried out, and a sample size calculation using an ANOVA for two groups indicates that a per-group sample size of 32 would permit detection of an effect size of 0.3 with 85% power. All data are mean ± SEM, and values of P < 0.05 were accepted as a statistically significant difference.

### Supplementary Information

Supplementary Information.

## Data Availability

The microarray raw data supporting the findings of this study are available in repository of Gene Expression Omnibus at https://www.ncbi.nlm.nih.gov/geo/query/acc.cgi?acc=GSE220219, accession number (GSE220219).
