# Supplementary material for: APOBEC-1 deletion enhances cisplatin-induced acute kidney injury
Source: Sci Rep. 2023 Dec 14;13:22255. doi: 10.1038/s41598-023-49575-3 (PMC10721635; doi:10.1038/s41598-023-49575-3)
Supplement: Supplementary file 1 — Supplementary Information. [file 41598_2023_49575_MOESM1_ESM.pdf]

APOBEC-1 deletion enhances cisplatin-induced acute kidney injury

Xiaojia Guo[1], Valerie Blanc[2], Nicholas O Davidson[2], Heino Velazquez[1,3], Tian-min Chen[1], Dennis G. Moledina[1,4], Gilbert W Moeckel[5], Robert L. Safirstein[1,3]*, Gary V. Desir[1,3]*

Supplemental Table S1. Characteristics of human participants included in the study

Characteristic	N=4
**Demographics**	
Age, years	73 (64, 81)
Female	3 (75%)
Black race	1 (25%)
Diabetes	0 (0%)
Hypertension	4 (100%)
Chronic kidney disease	4 (100%)
Body mass index (BMI), kg/m2	29 (27, 33)
**Baseline Laboratory Features**	
Baseline Creatinine, mg/dl	1.8 (1.5, 2.0)
Baseline GFR, ml/min	28 (27, 29)
Baseline Protein to creatinine ratio, mg/mg	0.4 (0.2, 3.5)
Baseline albumin to creatinine ratio, mg/mg	0.1 (0.0, 1.4)
**Features at Biopsy**	
Serum creatinine, mg/dl	3.4 (2.5, 3.7)
Blood urea nitrogen, mg/dl	64 (41, 92)

Median (interquartile range) or n (%) shown. No participants had any data missing except 1 had missing baseline protein to creatinine ratio.

Supplemental Figure S1

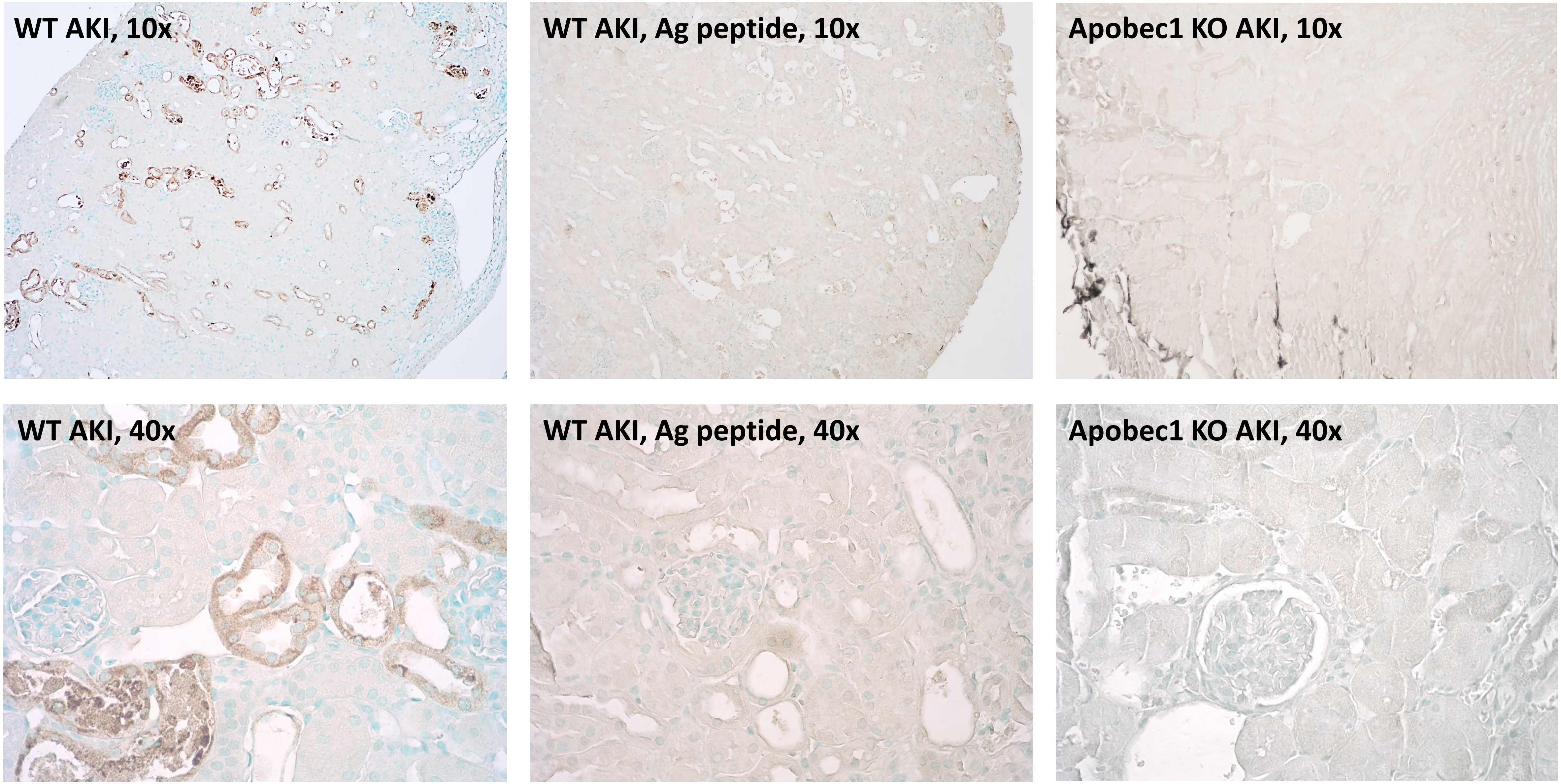

Supplemental Figure S1. Immunohistochemistry of Apobec1 in kidneys from WT or Apobec1 KO mice treated with CP for 4 days. Representative images of 10x (upper pictures) and 40x (lower pictures). Apobec1 positive staining (brown) was observed in tubules in WT AKI kidneys (pictures on the left), and these staining were blocked with 200x Apobec1 peptides used for generating the Apobec1 antibody (pictures in the middle). No positive staining was observed in Apobec1 KO kidneys (pictures on the right). Nuclei were stained with methyl green.

Supplemental Figure S2

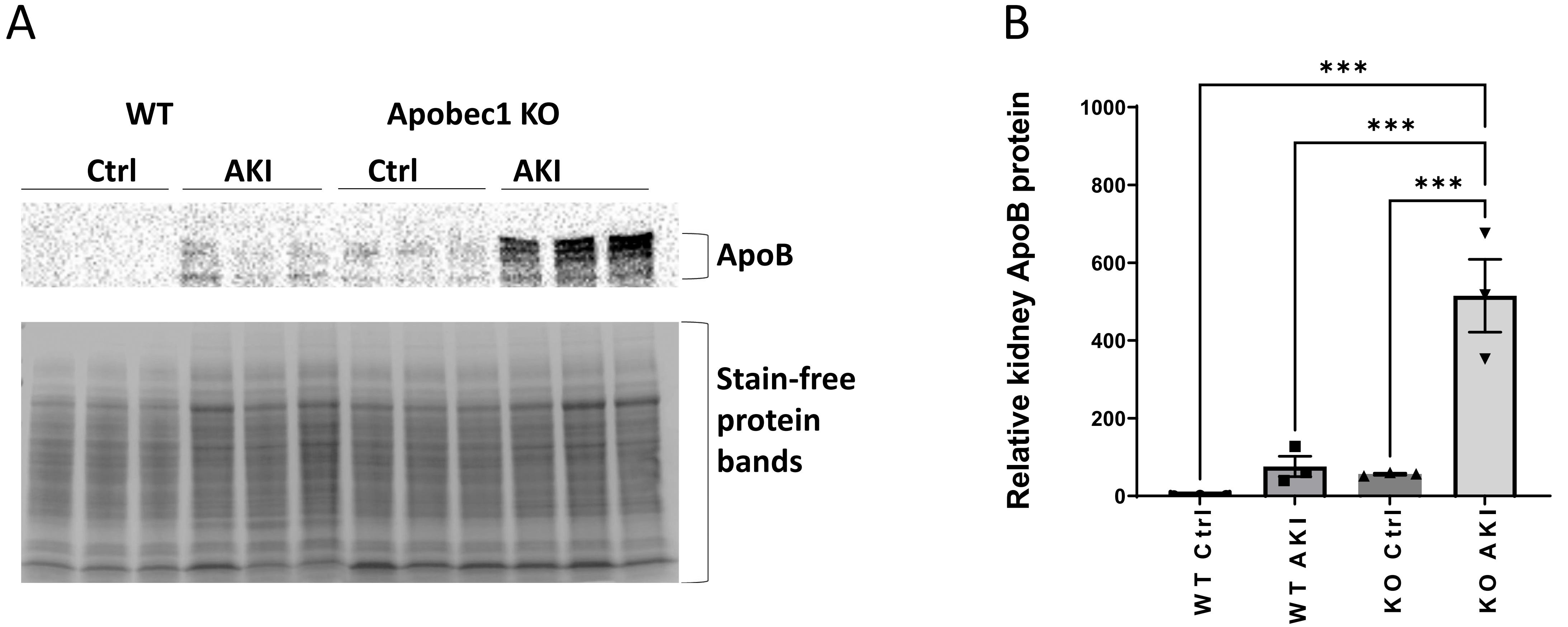

Supplemental Figure S2. Immunoblotting of ApoB in kidneys from WT or Apobec1 KO mice treated with CP for 4 days. A: A representative immunoblot for ApoB in kidney lysate (upper picture) and the image of the membrane after transfer (lower picture) for stain-free protein bands. B: Quantification of ApoB detected by immunoblotting and normalized by stain-free protein bands, one way ANOVA multiple comparison, n = 3, ***: $p<0.0005$.

Supplemental Figure S3

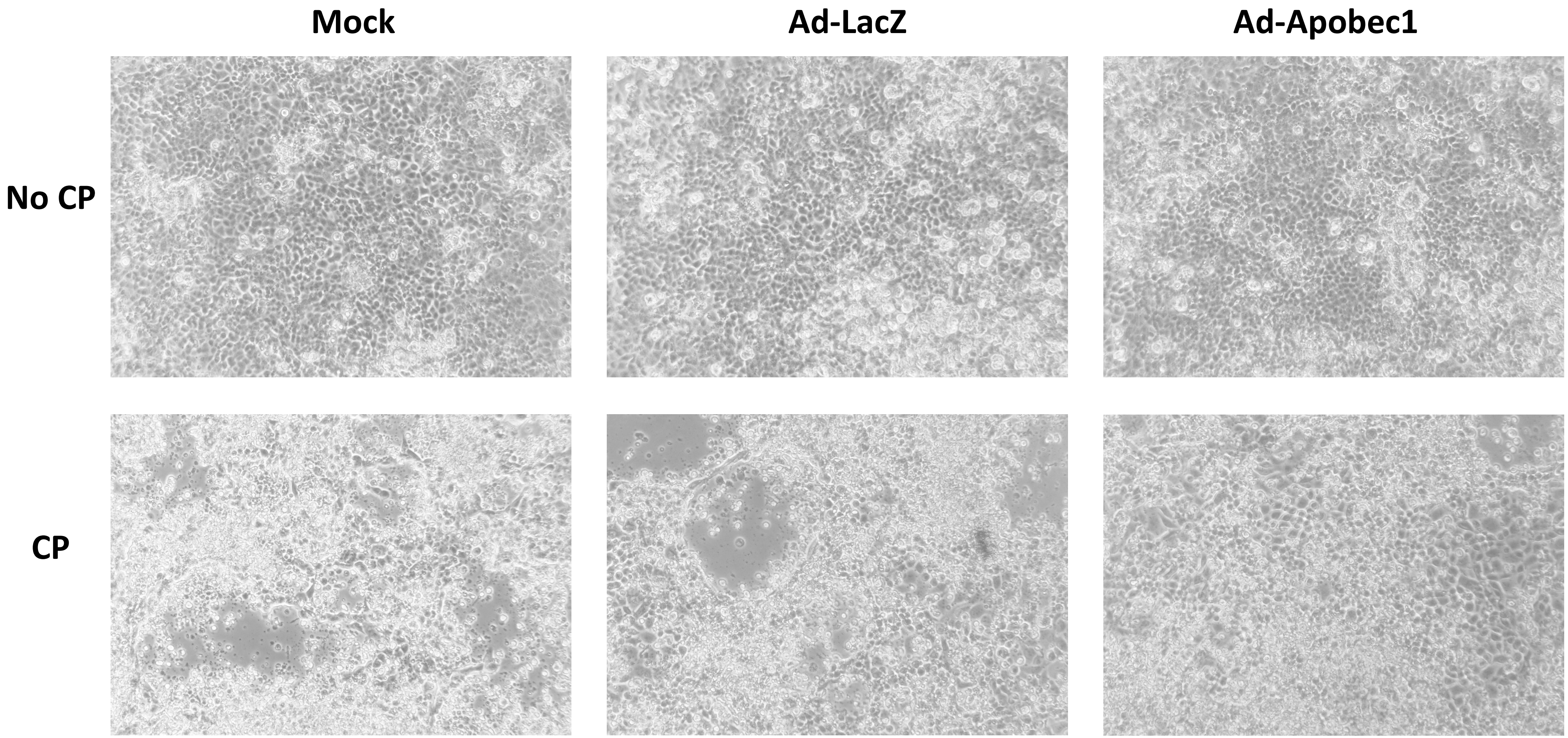

Supplemental Figure S3. Morphology of TKPTS cells transduced with adenovirus overexpressing Apobec1 or LacZ as a control at amount indicated for 2d, then treat with or without 25uM cisplating for 1d

Uncropped blot for Figure 2 I, immunoblot blotted for KIM-1

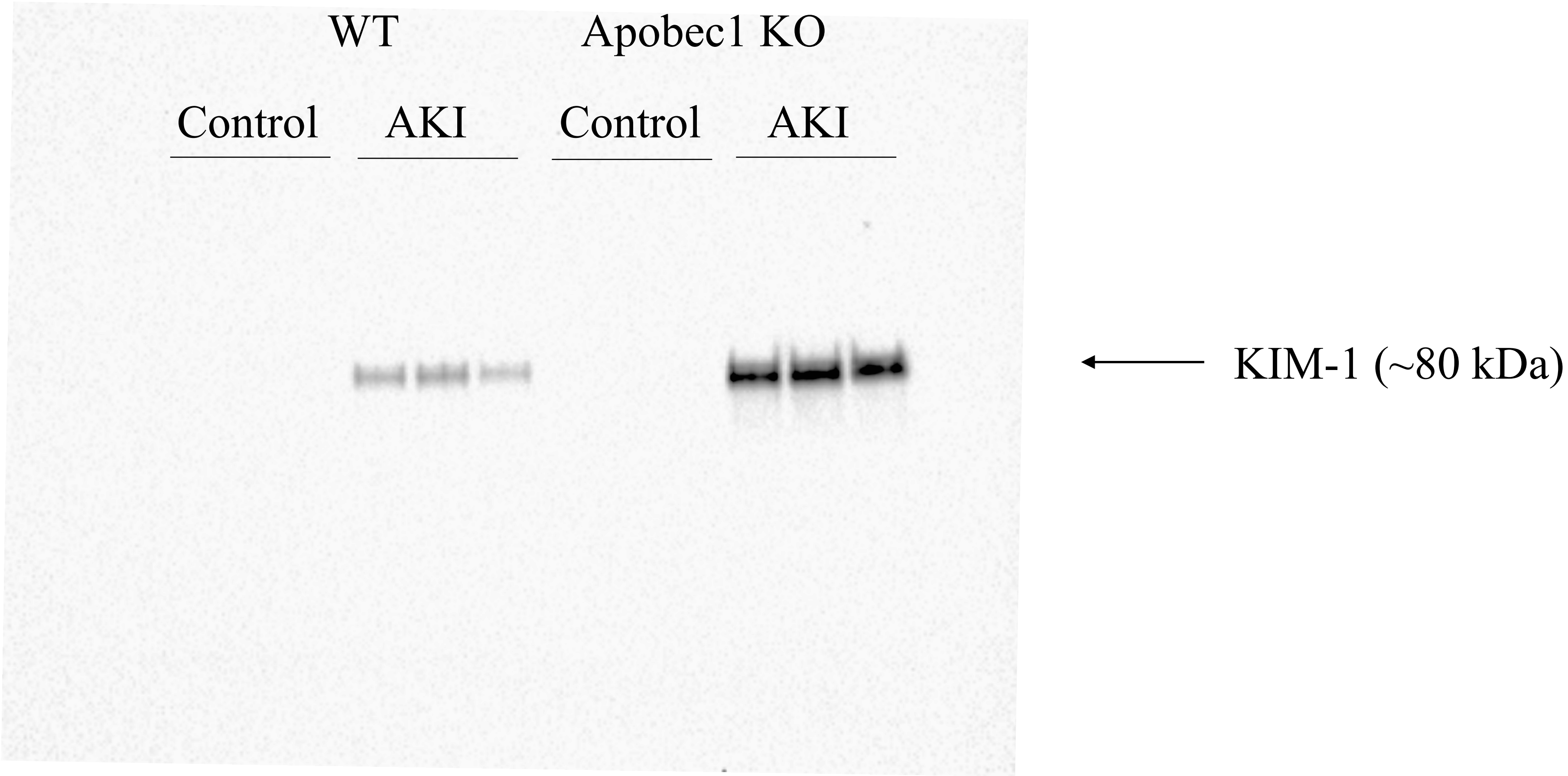

Immunoblot analysis of KIM-1 in kidneys from WT or Apobec1 KO mice treated with saline (Control) or cisplatin at 15 mg/kg for 4 days (AKI), n = 3 from each group.

Uncropped blot for Figure 2 I, immunoblot blotted for NGAL

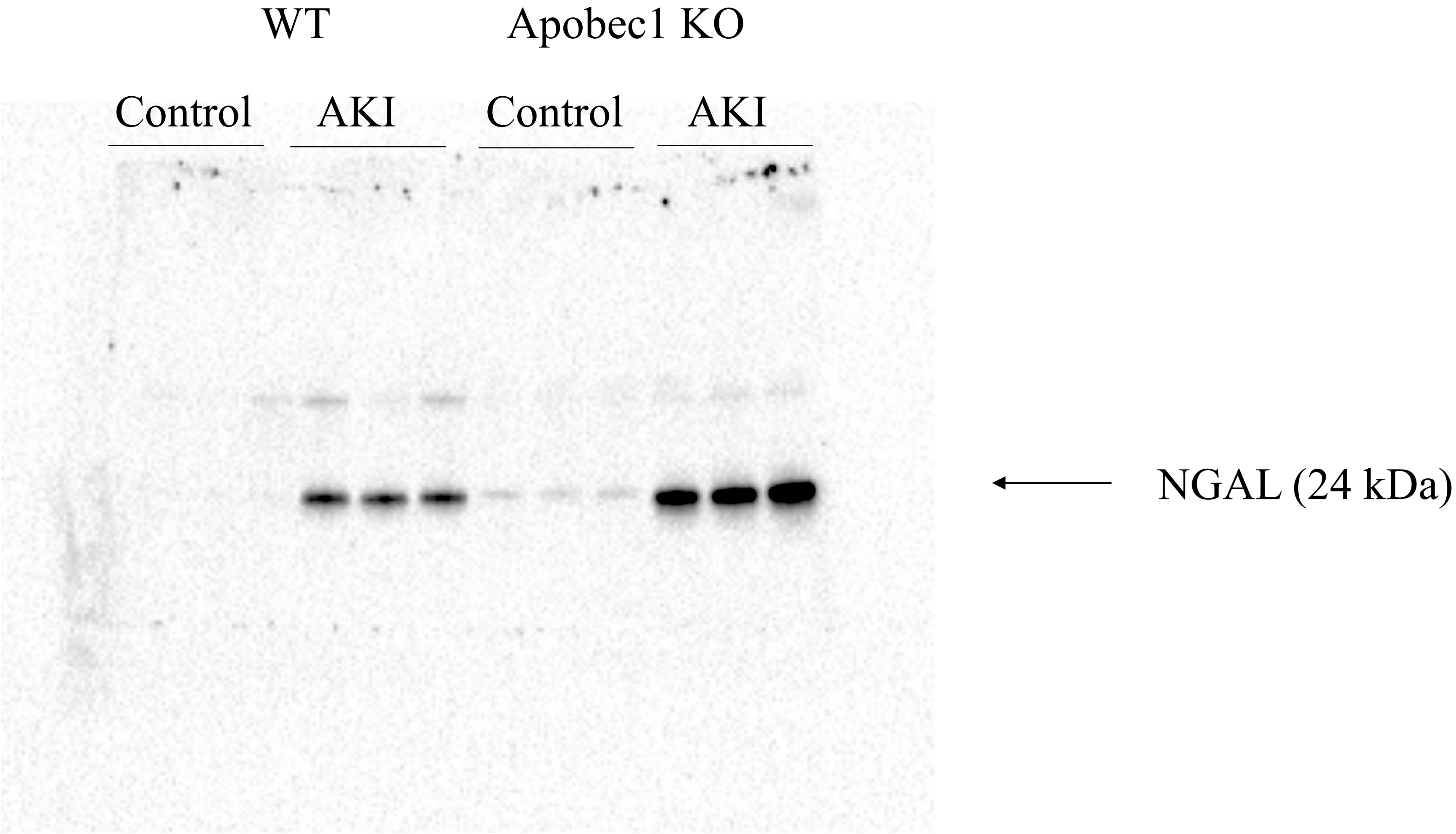

Immunoblot analysis of NGAL in kidneys from WT or Apobec1 KO mice treated with saline (Control) or cisplatin at 15 mg/kg for 4 days (AKI), n = 3 from each group.

Uncropped blot for Figure 2 I, immunoblot blotted for GAPDH

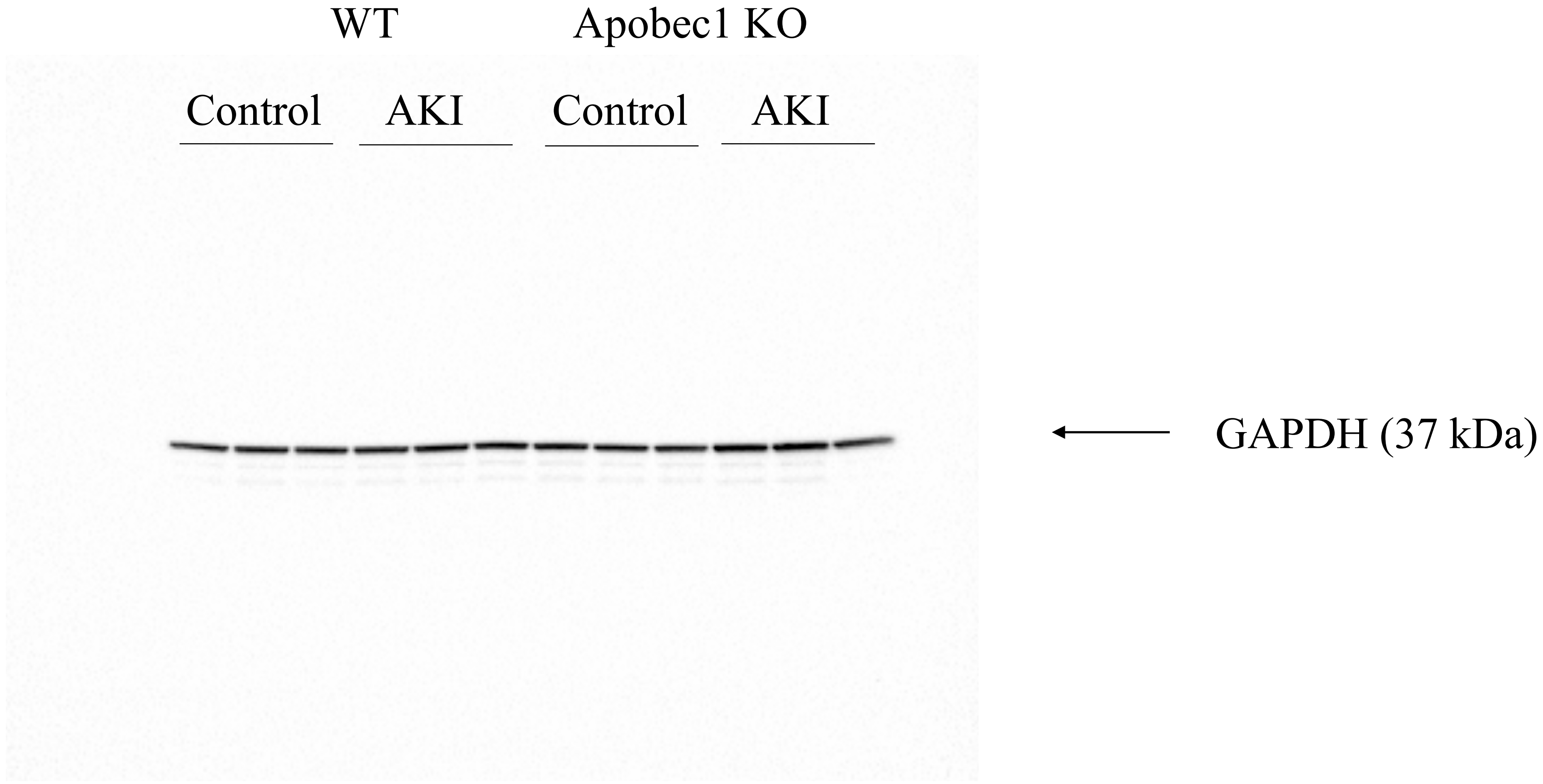

Immunoblot analysis of GAPDH in kidneys from WT or Apobec1 KO mice treated with saline (Control) or cisplatin at 15 mg/kg for 4 days (AKI), n = 3 from each group.

Uncropped blot for Figure 5 C, immunoblot blotted for serum ApoB

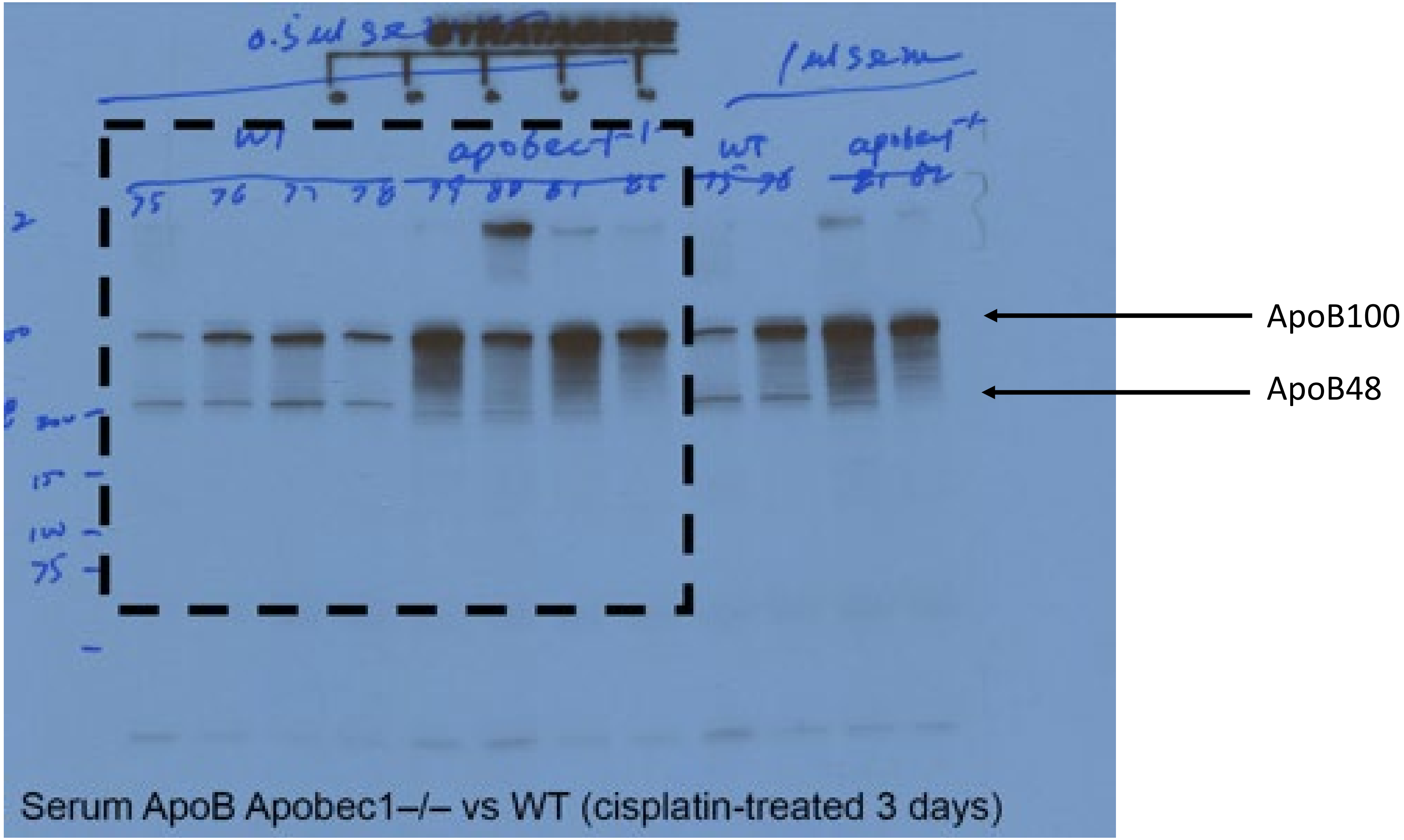

Uncropped blot for Figure 5 C, immunoblot blotted for kidney ApoB

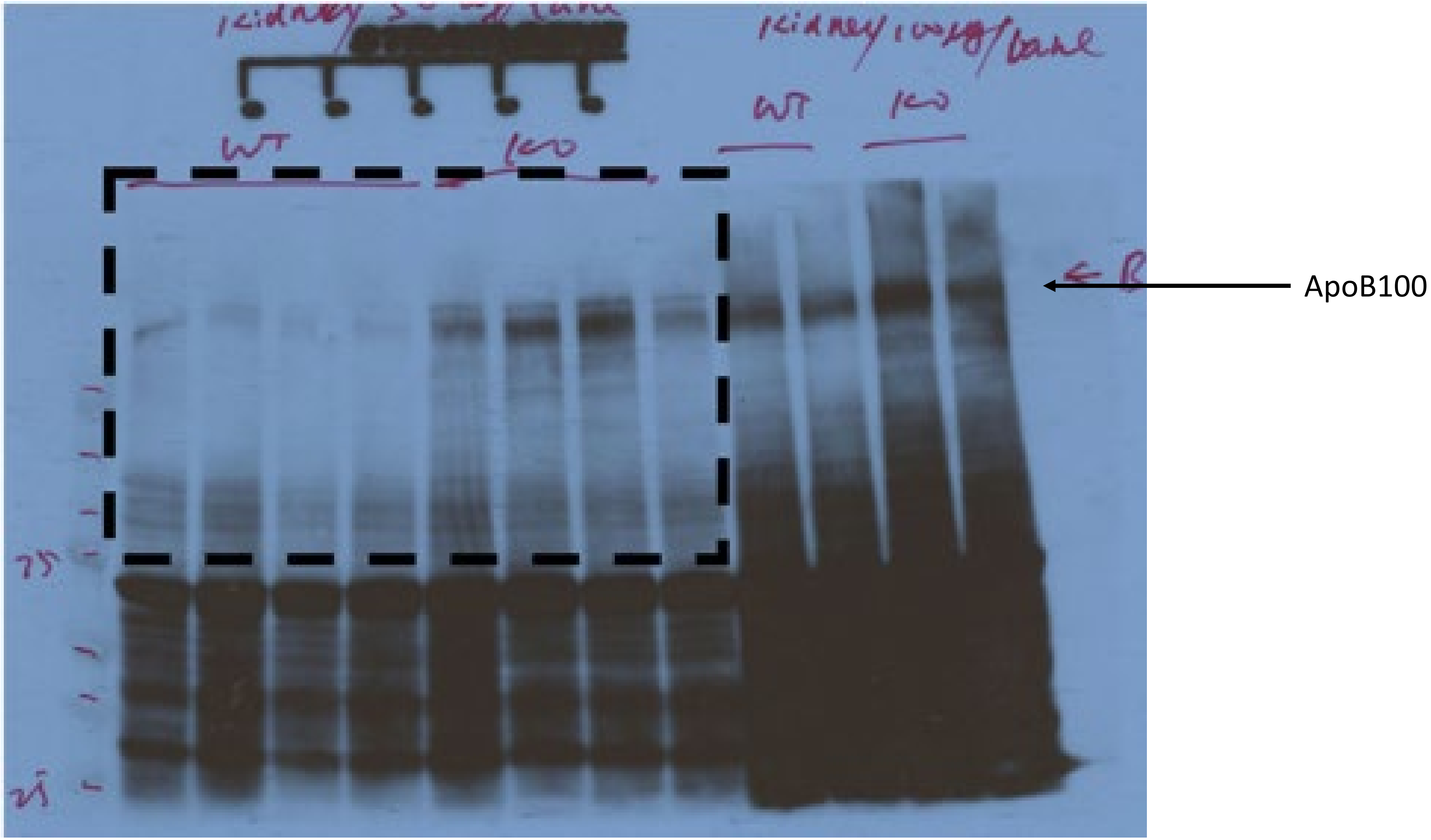

Uncropped blot for Figure 5 C, immunoblot blotted for kidney GAPDH

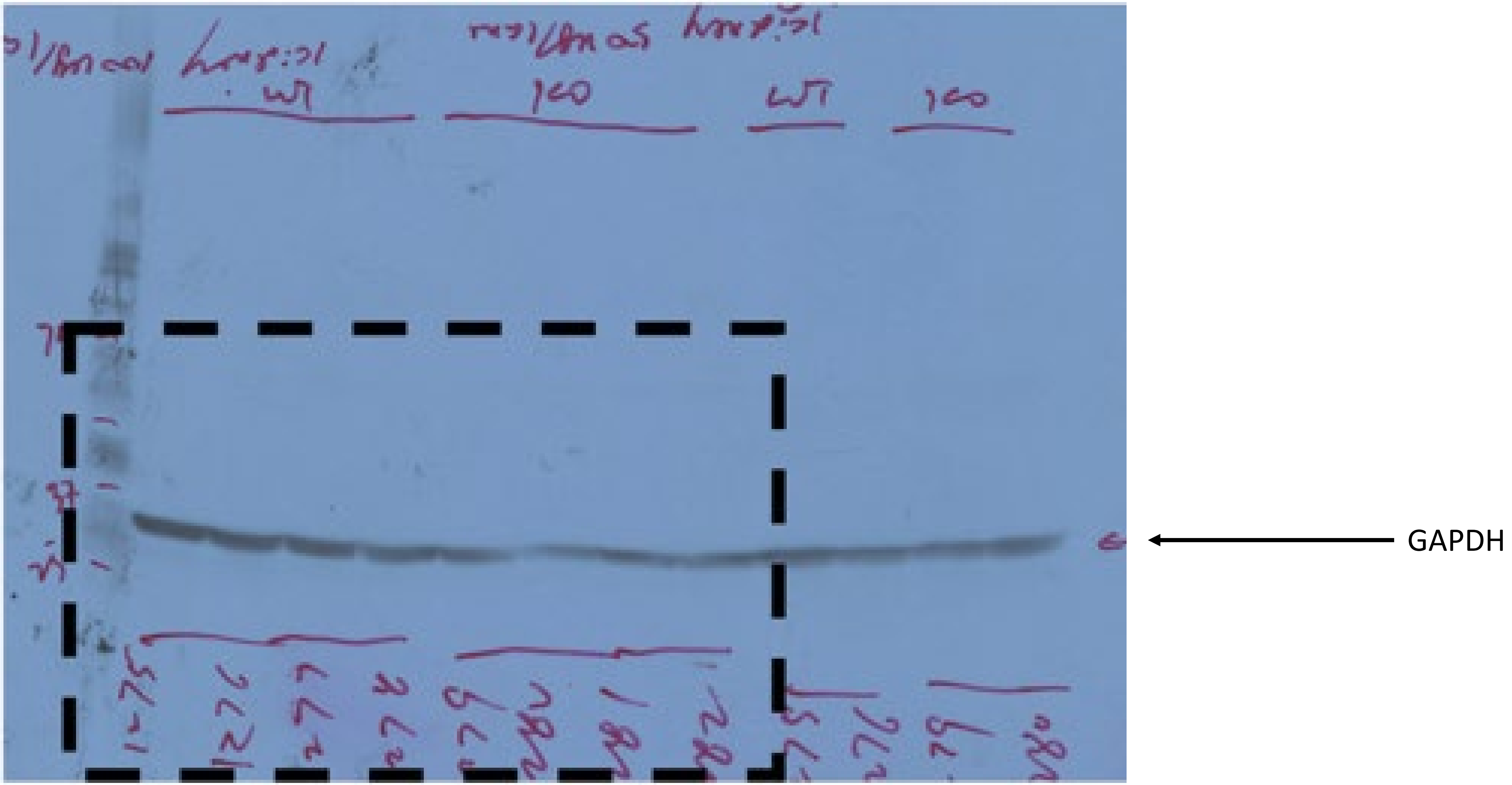

Uncropped blot for Figure 6 A, immunoblot blotted for Acsl4

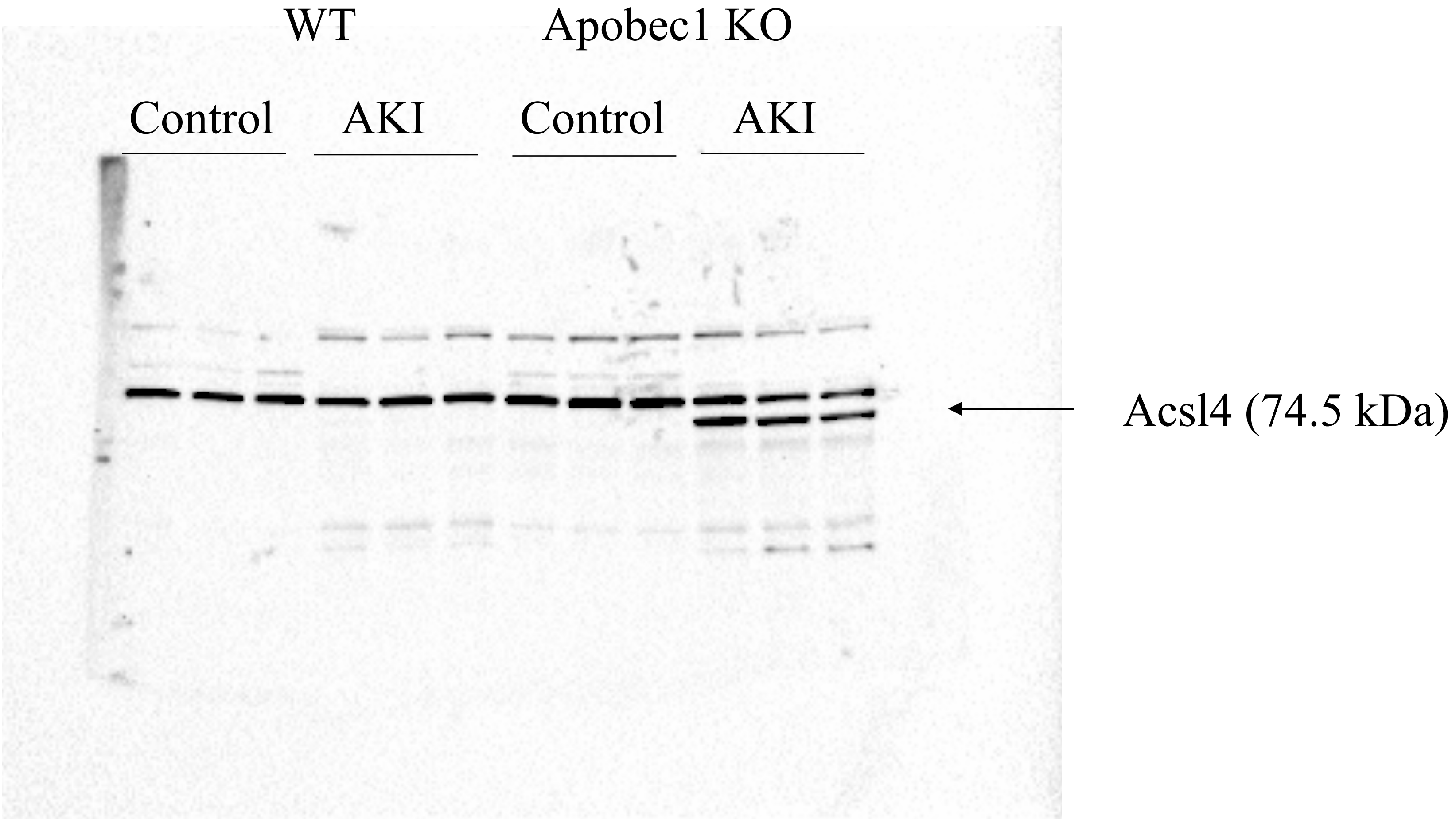

Immunoblot analysis of Acsl4 in kidneys from WT or Apobec1 KO mice treated with saline (Control) or cisplatin at 15 mg/kg for 4 days (AKI), n = 3 from each group.

Uncropped blot for Figure 6 A, immunoblot blotted for p-RIPK1

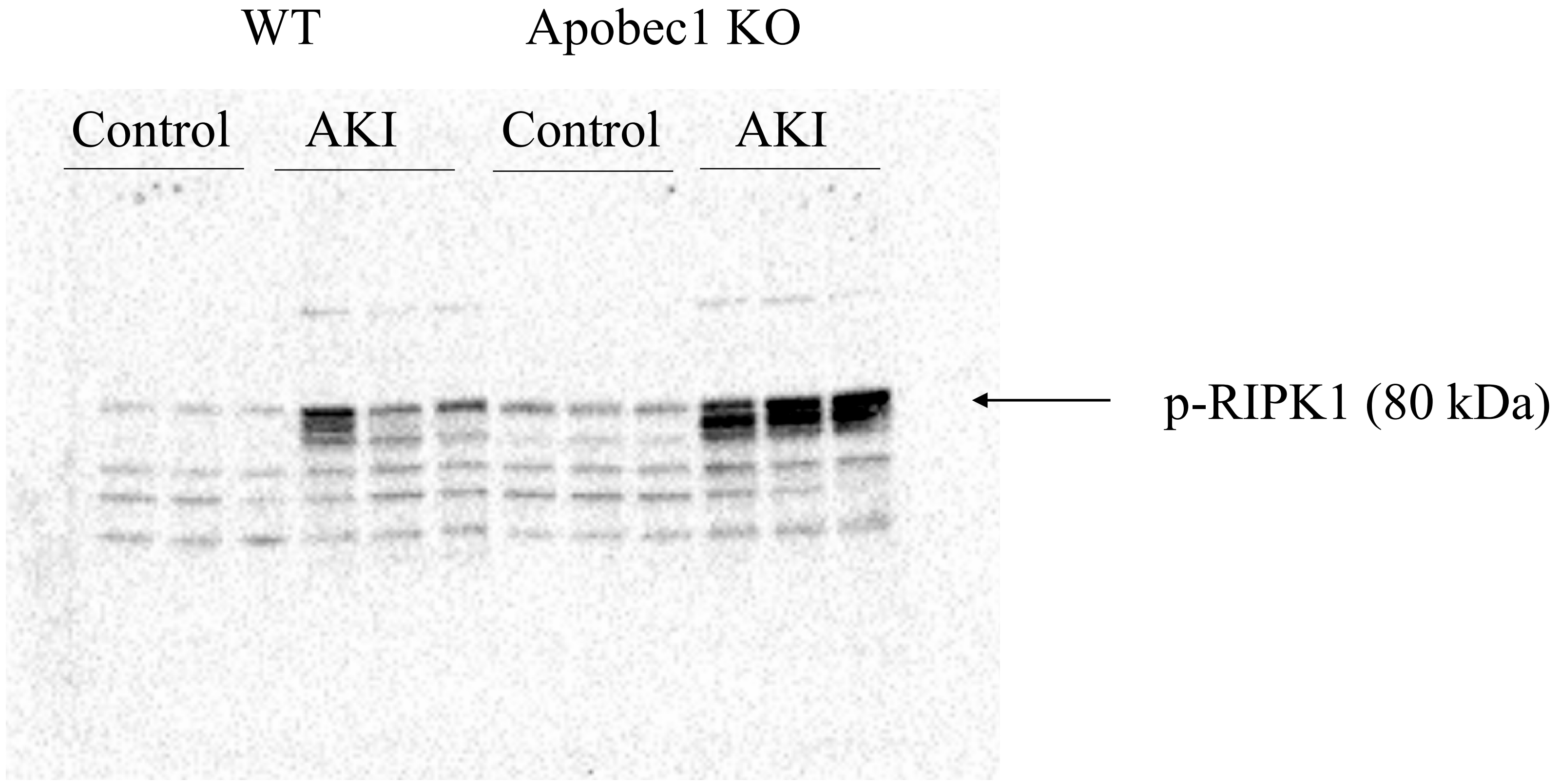

Immunoblot analysis of p-RIPK1 in kidneys from WT or Apobec1 KO mice treated with saline (Control) or cisplatin at 15 mg/kg for 4 days (AKI), n = 3 from each group.

Uncropped blot for Figure 6 A, immunoblot blotted for GAPDH

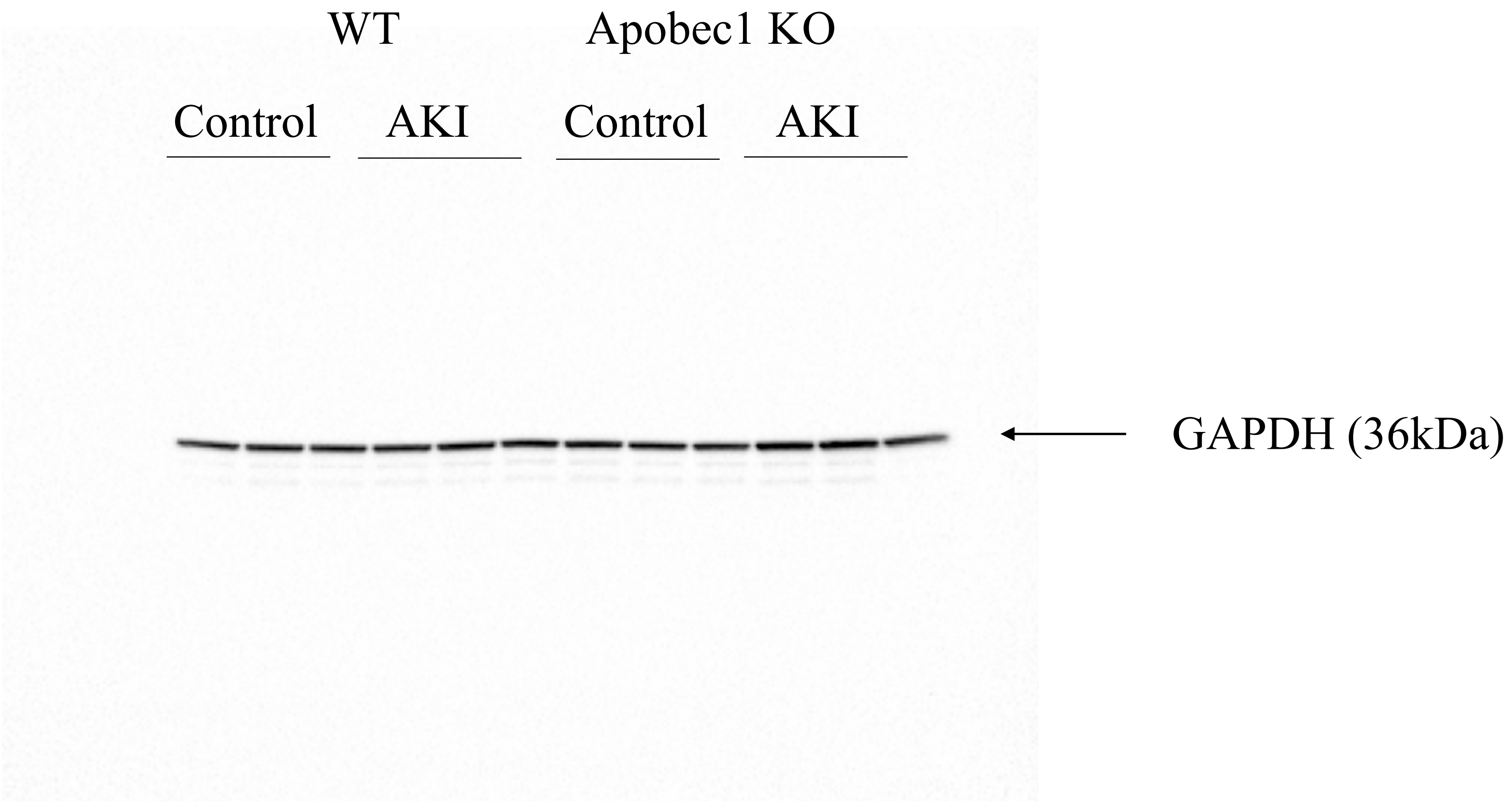

Immunoblot analysis of p-RIPK1 in kidneys from WT or Apobec1 KO mice treated with saline (Control) or cisplatin at 15 mg/kg for 4 days (AKI), n = 3 from each group.

Uncropped blot for Supplemental Figure S2, immunoblot blotted for ApoB

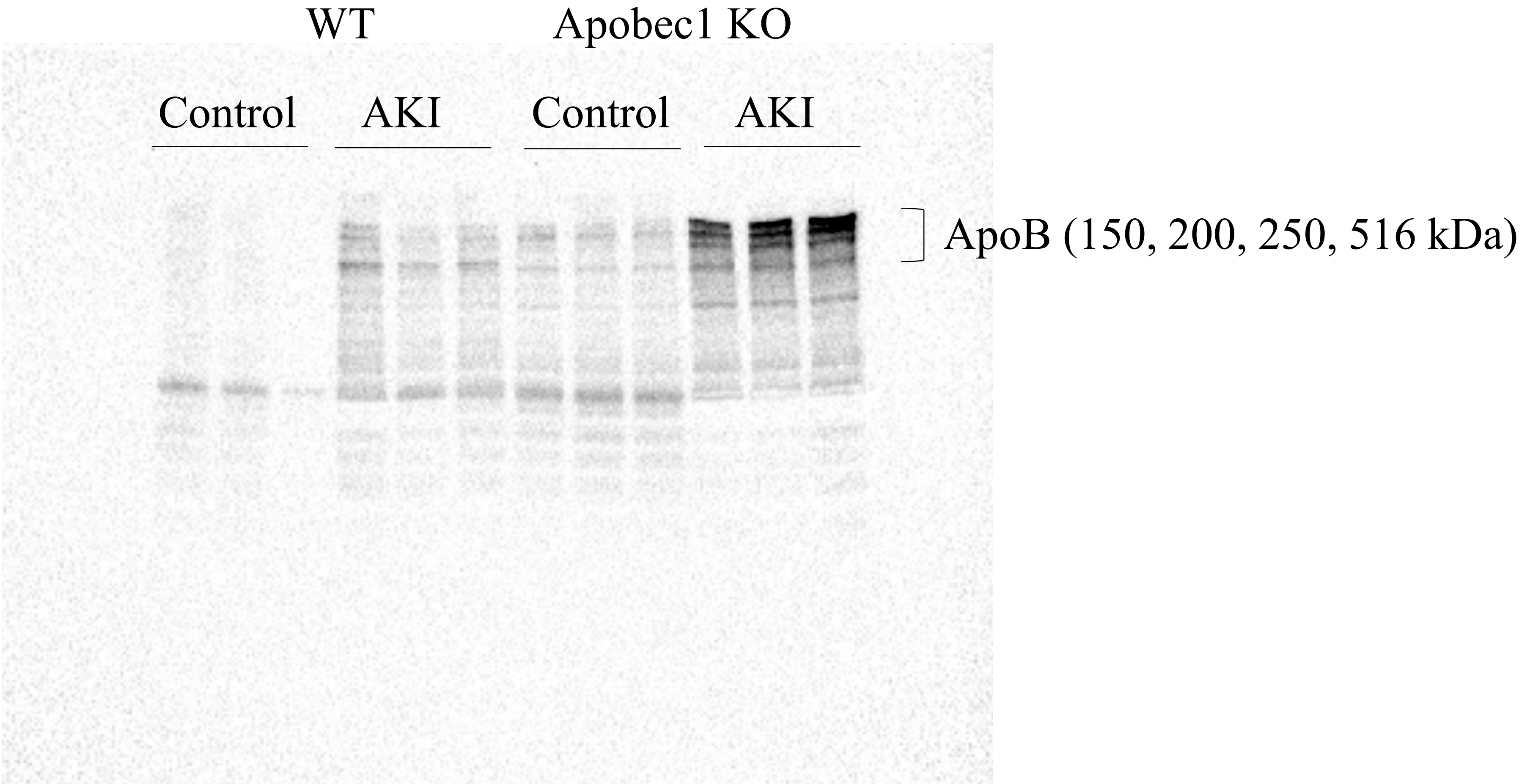

Immunoblot analysis of ApoB in kidneys from WT or Apobec1 KO mice treated with saline (Control) or cisplatin at 15 mg/kg for 4 days (AKI), n = 3 from each group.
